# Digital occlusal analysis of pre and post single posterior implant restoration delivery: A pilot study

**DOI:** 10.1371/journal.pone.0252191

**Published:** 2021-07-02

**Authors:** Ting Zhou, Jirapa Wongpairojpanich, Maytha Sareethammanuwat, Charukrit Lilakhunakon, Borvornwut Buranawat

**Affiliations:** 1 Department of Periodontics & Implant Dentistry, Faculty of Dentistry, Thammasat University, Phatum Thani, Thailand; 2 Department of Orthodontics, School of Stomatology, Kunming Medical University, Yunnan, China; Danube Private University, AUSTRIA

## Abstract

**Objectives:**

The purposes of this study were to analyze the effects of single posterior implant restorations delivery on the redistribution of bite force and to evaluate the changes in occlusal force distribution of prostheses and potential influencing factors on occlusion variation at different stages.

**Materials and methods:**

Thirty-two single posterior restorations in 30 participants (18 women and 12 men aged 27 to 75 years) were placed into either a unilateral single-tooth defect (n = 17) or on either side of a bilateral teeth defects (n = 15). The bite force (%) of the prostheses, teeth and segments at the maximum intercuspation position (MIP) was evaluated using a T-scan at 5 stages (pre-placement, immediately following placement, and 2 weeks, 3 months, and 6 months post-placement).

**Results:**

The occlusal force of implant-supported prostheses was significantly (*P* = .000) lower than those of the control natural teeth at the baseline, then no significant difference was found with that of the mesial teeth at 3 months, and finally it was significantly (*P* = .000) lower than that of the distal teeth at 6 months; meanwhile, it significantly (*P* = .008) increased by a mean of 2.04 times from 2 weeks (3.39 ± 2.61%) to 3 months (6.90 ± 4.77%), whereas no significant difference (*P =* .900) was found from 3 months (6.90 ± 4.77%) to 6 months (7.31 ± 4.60%). In addition, the bite force of the posterior segment on the restored side of both unilateral and bilateral gaps was significantly (*P* = .013,.001) improved by 3.31% and 6.83%, respectively, although the discrepancy in bite force significantly (*P =* .039) increased from an initial 3.52% to 5.02% for subjects with bilateral defects, accompanying increases in the proportion (15.38%) of the level III bilateral bite force deviation (*P >*.05).

**Conclusions:**

Bite force and masticatory ability can be improved with the immediate delivery of a single posterior implant restoration. The bite force distributed on the implant prosthesis inevitably increases after placement of implant prostheses, a routine follow-up and occlusal evaluation are strongly needed.

## Introduction

The occlusion of dental implants has characteristics inherently similar to natural and restored dentitions, but the biophysiological differences between a tooth and an implant make it impossible to apply the natural teeth occlusion concept to dental implants directly. Compared with natural dentition supported by the periodontal ligament (PDL), the displacement of osseointegrated dental implants has been reported to be approximately 3–5 μm vertically and 10–50 μm laterally, while the values of natural teeth are 25–100 μm and 56–108 μm, respectively [1–3]. In addition, dental implants exhibit low tactile sensitivity and proprioceptive motion feedback because of the absence of periodontal mechanoreceptors [4]. It is believed that dental implants may be more prone to occlusal overloading, so implant-protected occlusion (IPO) [5] has been attempted to reduce biomechanical stress at the implant interface and prosthesis [6].

For single implant protheses, the force distribution should be equal bilaterally and maximized on adjacent teeth [1, 7], with light contacts for heavy bites and no contact for light bite at the maximum intercuspation position (MIP) [7]. Thus, along with implant restorations, the distribution of occlusal forces on adjacent teeth and dental arches are also worthy of attention, to avoid overload resulting from an improper occlusion [8] including a poor force distribution. However, whether the occlusion of implant prostheses remains in “light contact” is unknown [9] due to objective existences of occlusal surface wear [10, 11], continued eruption of opposing teeth and mesial shift of the natural teeth [12, 13]. In addition, the goals of implant occlusion involve the creation of a functionally effective masticatory scheme that minimizes occlusal loads. The delivery of implant prostheses has a positive effect on the maintenance of the functional tooth unit (FTU), which is defined as two opposing teeth occluding each other [14, 15]. As the smallest possible increment of FTUs, due to the“light contact”characteristic, coupled with the patient’s personal anatomy as well as the complexity of the occlusion, the effect of a single posterior dental implant-supported restoration loading on the distribution of occlusal force remains questionable, and the stability of this new occlusion constructed by implant prosthesis insertion is still in doubt [16]. Current studies are either in vitro or animal experiments, or mainly clinical retrospective studies and case reports [17], objective and quantitative clinical trials and evidences support are lacking [18, 19].

In general, clinical occlusion is primarily determined with articulating papers and shim- stock foils. First, the mark size of articulating paper has been shown to be an unreliable indicator of occlusal force [20, 21] and inadequate for interpreting the occlusal load [22]. Next, none of the conventional methods, such as the use of articulating paper, shim-stock foil, and impression waxes, are able to quantify occlusal contacts. Digital methods, such as T-scans, not only record the instantaneous occlusal contact, including the position, intensity and distribution in the chewing cycle to the nearest 0.01 seconds dynamically, but also objectively reflect the distribution and changes in the occlusal contact for each tooth and dental arch [23, 24]. Furthermore, the trajectory of the center of occlusal force (COF) obtained from a T-scan can assist determining the dynamic change in the balanced occlusal force. This provides a quantitative, objective and reliable method for the analysis of "dynamic" occlusion [25, 26].

The purposes of this study were to analyze the effect on the redistribution of bite force following crown delivery via digital measurements and to evaluate the changes in occlusal force distribution of single posterior dental implant restorations and possible influencing factors on occlusion variation at different stages. The null hypothesis was that the implantation of single posterior restorations could not bring about a significantly increased bite force in the posterior segment of the restored side when immediately following placement, and the occlusal force of implant restorations would not change significantly within a short period of 6 months.

## Materials and methods

**Fig 1:**
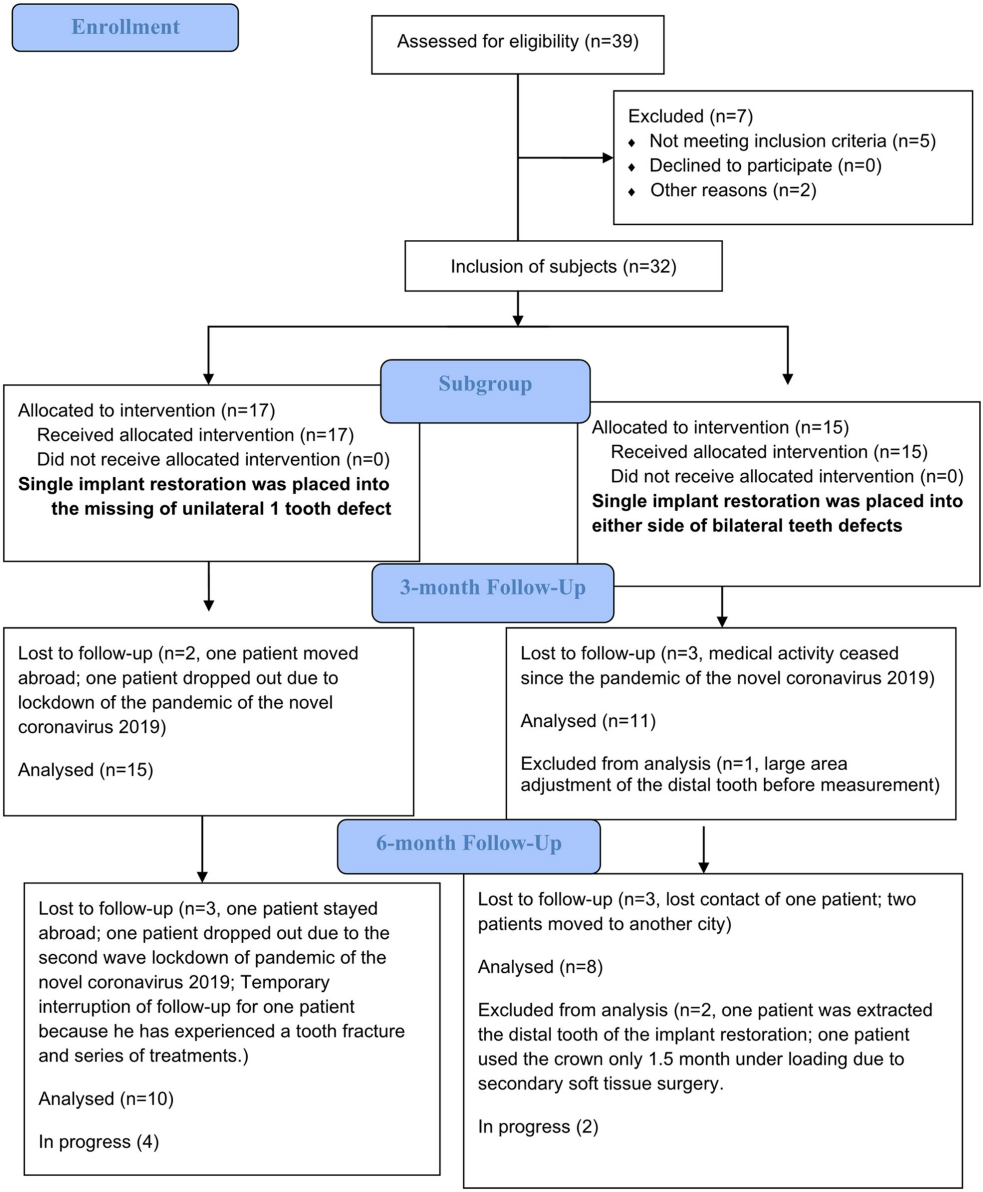
Research flow chart.

This prospective study included 5 phases of occlusal measurement, including before crown delivery, immediately after insertion, at 2-week, 3-month and 6-month follow-ups. The study design was approved by the Ethics Committee of Thammasat University (approved number: 135/2562, date: 11/10/2019), and registered in the Thai Clinical Trials Registry (approval number: TCTR20200909004, date: 09/09/2020). Patients who received a posterior single-implant restoration at the Faculty of Dentistry, Thammasat Hospital were enrolled in this study from October 2019 to December 2020. Data regarding patient age, sex, current medical and dental problems, parafunctional habits, periodontal tissue status, region of implantation, length and diameter of implant, crown material and opposing teeth, were recorded. The study was conducted in accordance with the Declaration of Helsinki and prospective research guidelines (Fig 1), and signed informed consent forms were obtained from patients before the study began. The clinical trial registration was delayed after obtaining ethical approval, this due to the authors were initially define this occlusal measurement as a routine clinical procedure with minimal risk to patients. However, immediately after informed by Thai Clinical Trials Registry, all authors confirm that all ongoing and related trials for this intervention were registered.

### Sample size calculation

The sample size was calculated according to the experimental research method, and the analysis index was “the change in the bite force distribution with a single posterior implant restoration pre- and post-insertion,” assuming that bite force was the data to be measured [16, 27]; an average increase of 2 units of bite force after insertion was considered to be significant if the standard deviation of the difference in the bite force for the subjects in the study was σ = 3 units, α was 0.05 on both sides, and the test efficiency 1-β was 0.90. Then, the standard deviation of the sample bite force s = (σ =) 3 units, δ = 2 units of bite force change, α = 0.05, and Zα2 = 1.96, β = 0.10, *Z*_*β*_ = 1.282 were calculated to obtain 23.65, that is, at least 24 subjects needed to conduct pre- and post-experimental comparisons. The formula for calculating the sample size is as follows:

n=Zα2+ZβSδ2

### Inclusion and exclusion criteria

The inclusion criteria were as follows: (1) age, 18–75 years old; (2) a single posterior dental implant restoration in which the adjacent teeth were natural; (3) successfully completed osseointegration in the implanting area; (4) in occlusion with natural dentition, i.e., the opposed teeth were natural teeth or nonimplant supported prostheses; and (5) there was only one posterior tooth missing space in the dentition, or if more than one tooth was missing, only a single implant was restored. The exclusion criteria were as follows: (1) severe temporomandibular joint disorders (one or more of the following issues: severely impaired mobility of the mandible, pain of 2 or more sites during movement of the mandible, locking or luxation of the joint, tenderness to palpation in 4 or more sites in muscle, limited mouth opening < 2.5 cm); (2) use of known drugs that would affect the central nervous system or severe systemic diseases or known mental disorders; (3) anterior open occlusion; (4) history of bruxism (teeth clenching or grinding during night sleep, accompanied by one or more of the following symptoms: discomfort or pain in the jaw muscle after waking up, abnormal teeth wear, masseter muscle hypertrophy when teeth are clenched, tooth sensitivity to cold and heat, gum recession, and joint noise during palpation of the temporomandibular joint); and (5) unwillingness to participate in a further follow-up.

### Acquisition of occlusal parameters

On the visit day, prior to crown delivery, the T-scan sensor III (Tekscan, South Boston, USA) was placed in the patients’ mouths, and the patient was instructed to bite forcefully at the maximum intercuspation position (MIP) three times. Later, the responsible doctors used articulating paper (40 μm thick, Nordin, Switzerland) and shim-stock foils (8 μm thick, Coltene, Germany) to make occlusal adjustments then obtain the clinically recognized “light contact” status under the guidelines for a routine occlusal examination: obviously shallower and smaller mark sizes compared with the adjacent teeth, shim-stock foils was easier to pass the interocclusal gap of prosthesis and antagonistic tooth when heavy biting, light contacts at a heavy bite and no contact at a light bite in MIP, canine guidance or anterior guidance in eccentric excursions. Then, the digital sensor was used again by the same examiner, and occlusion was recorded on the MIP 3 times. The process was repeated 2 weeks later at final torque, and 3 months and 6 months after implant restoration placement. Calibration of sensor sensitivity was completed before each recording. The measurement of T-scan at 2-week was completed after screw hole resealing due to final torque used, but that of 3-month and 6- month was applied before any occlusal adjustments of prosthesis.

The width of each tooth was measured and imported into the T-scan software to ensure accurate tooth position, for rotated teeth or scattered spaces, recording the actual occlusal table width. The bite forces of each tooth and 6 segments (left and right anterior, left and right posterior, left and right half) are displayed as a percentage in the T-scan image. The color of the contact area shown in red (orange), green, and dark blue correspond to high, moderate, and low densities of bite force, respectively. A proper occlusal adjustment was made when a high-density spot (red) was found. The dynamic image showed the sequences and changes in the force and contact area with time from the first tooth contact to the stable maximum intercuspation position and, finally, disoccluded teeth, and the average of 3 measurements were used for analysis; the center of occlusal force (COF) was also recorded.

### Statistical analysis

A commercially available software program, SPSSAU (version 20.0, Beijing, China, online application software, https://www.spssau.com), was used for analysis. A *P*-value <0.05 was considered statistically significant. The intragroup correlation coefficient (ICC) of the bite force of posterior segments immediate following insertion and implant restorations at 3 months and 6 months was tested. Descriptive data, including the mean ± standard deviation and median, were used to express the bite force distribution in restorations, teeth and segments, and the Shapiro-Wilk test was used to detect whether the quantitative data were normally distributed. The Wilcoxon rank sum test, Kruskal-Wallis test and Dunn test were used for comparisons of the bite force distributed on implants and teeth. The Wilcoxon rank sum test, and Mann-Whitney U test were used for comparisons of the bite force distributed on segments. The Kruskal-Wallis test and Dunn test were used to assess the changes in the bite force distributed on the implant and teeth at different times. The Friedman test and Nemenyi test were used to assess the changes in the bite force distributed on the implant and teeth over time. The Pearson χ2 test was used for comparison of the bilateral bite force deviation and location of the occlusal force center. A Stepwise regression test was used for analysis of influencing factors, including demographics and implant characteristics, on the bite force distribution of implant restorations. The correlation between the length/diameter and the force of implant was assessed with the Spearman test.

## Results

Thirty patients (18 female and 12 male aged 27 to 75 years) were included in the study. Two patients received a second crown on the contralateral side 3 months after placement of the first crown. No implant or components failures were found during the observed periods. To ensure sufficient and reliable data analysis, patients with available data were divided into 2 groups based on the targeted research purposes because the novel coronavirus 2019 pandemic had an impact on increasing the loss of follow-up and time. Group 1 was recorded pre-treatment and immediately post-placement of 26 participants (14 female and 12 male aged 32 to 75 years); group 2 included 24 (15 female and 9 male aged 27 to 75 years) and 18 patients (11 female and 7 male aged 43 to 75 years) who completed the 3-month and 6-month follow-up, respectively. No obvious alternations occurred in the 3-month follow-up, such as implantation of new fixed restorations, large-area filling or extraction of teeth compared, or other conditions that might affect the results; while the new implant-supported crown had been placed in 5 patients among the 18 patients who have completed 6 months follow-up. Another two patients completed the follow-ups, but the data were excluded from the analysis because of extraction of the distal tooth or actual short loading period (S2 File). The demographic information is summarized in Table 1, and the details of the implants and prostheses are listed in Table 2. In addition, the ICCs mentioned above were 0.982 (95% CI: 0.962–0.992), 0.961 (95% CI: 0.920–0.982) and 0.899 (95% CI: 0.779–0.959), respectively.

**Table 1:** Patients’ demographic information.

	Group 1 Pre and immediate insertion		Group 2 Post crown insertion (completion of follow-ups)	
	Pre	immediately	3-month	6-month
**Total (male/ female)**	26 (12/14)		24 (9/15)	18 (7/11)
**Mean age(min/max)**	54.20 ± 9.77 (32/75)		54.63 ± 10.96 (27/75)	55.83 ± 9.46 (43/75)

**Table 2:** Details of the implants and prostheses.

	Group 1 Pre and post crown insertion		Group 2 Post crown insertion (completion of follow-ups)	
	Pre	immediate	3-month	6-month
**Total (premolar/molar)**	27 (4/23)		26 (3/23)	18 (3/15)
**Average follow up time (month)**			4.17 ± 0.83	8.42 ± 1.62
**Location**				
upper (premolar/molar)	11 (4/7)		8 (3/5)	6 (3/3)
lower (premolar/molar)	16 (0/16)		18 (0/18)	12 (0/12)
**Materials of crown**				
zirconia/others	26/1		26/0	18/0
**Opposing teeth**				
natural / crown/ amalgam filling	20/5/2		19/5/2	13/3/2
**Category**				
category 1 unilateral tooth defect (one missing)				
initial FTU (restored<contralateral side)	14			
category 2 bilateral teeth defects (more than one missing)				
initial FTU (restored = contralateral side)	8			
initial FTU (restored>contralateral side)	3			
initial FTU (restored<contralateral side)	2			
**Retention**				
screw retained/cement retained	26/1		26/0	18/0
**Diameter(mm)**				
narrow(<3.75)/ standard (3.75–5)	1/26		1/25	1/17
min/max	3.3/5.0		3.3/5.0	3.3/5.0
**Length(mm)**				
short (≤6) /standard (>6)	3/24		2/24	1/17
min/max	6.0/10.0		6.0/10.0	6.0/10.0

### Bite force exerted on implant-supported prostheses and natural teeth

**Fig 2:**
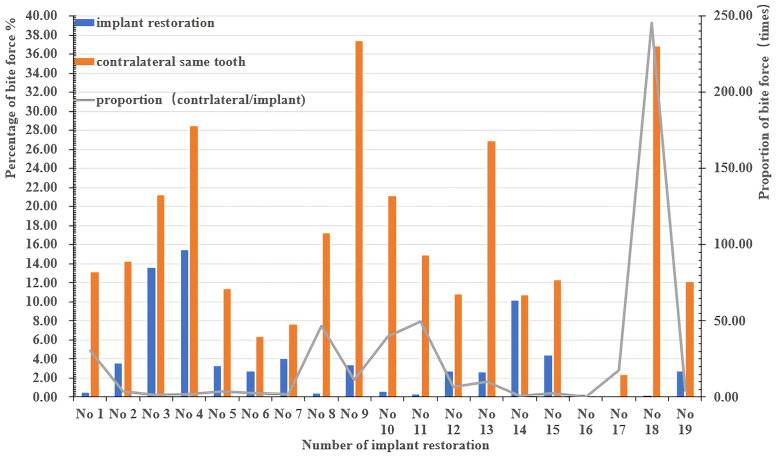
Percentage and proportion of the bite force between contralateral same teeth and implant restorations (immediately following crown insertion).

**Fig 3:**
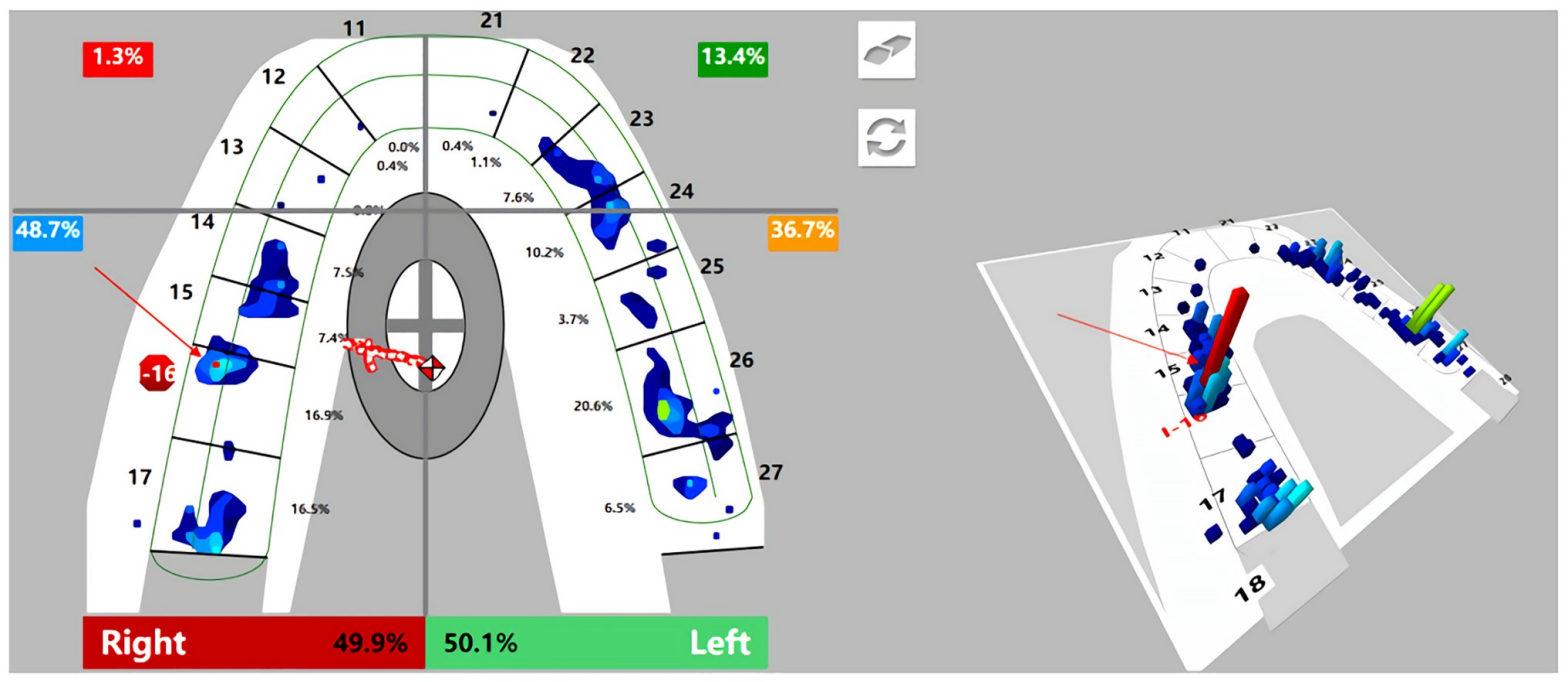
High density of the bite force on implant crown (16) from T-scan (inserted immediately). Red arrows indicate 2D and 3D images of the high density of occlusal contact.

After placement of a single posterior implant restoration, the changes in the bite force distributed on mesial, distal and contralateral teeth were not significant (*P* = .485,.627,.896, respectively). Compared with natural teeth, the occlusal force of implant prostheses was significantly smaller than that of mesial, distal and contralateral teeth (*P* = .000) (Table 3). Large proportions and high densities of bite force were found (Figs 2 and 3).

**Table 3:** Comparison of the bite force distributed on implants and teeth (before treatment vs immediately following restoration).

	Implant	Mesial	Distal	Contralateral	*P* value
	n = 27	n = 27	n = 23	n = 19	
**Group 1 27 implant restorations in 26 patients**					
**Pre**	/	10.12 ± 8.34 (7.650)	18.98 ± 12.04 (17.200)	16.33 ± 9.40 (16.230)	0.009**
**Immediately**	4.39 ± 4.28 (3.270)	10.06 ± 10.70 (6.470)	18.25 ± 10.27 (17.530)	16.03 ± 10.39 (13.070)	0.000**
***P* value**	/	0.485	0.627	0.896	

Note:

* *P* < 0.05;

** *P* < 0.01;

Descriptive data: mean ± standard deviation and (median).

Pre: distal *>* mesial (*P* = 0.004 < 0.01); contralateral *>* mesial (*P* = 0.024<0.05); Immediately: distal *>* implant (*P* = 0.000 < 0.01); contralateral >implant (*P* = 0.000 < 0.01); distal *>* mesial (*P* = 0.004 < 0.05); mesial *>* implant (*P* = 0.016 < 0.05); contralateral *>* mesial (*P* = 0.047 < 0.05).

### Redistribution of occlusal force

**Fig 4:**
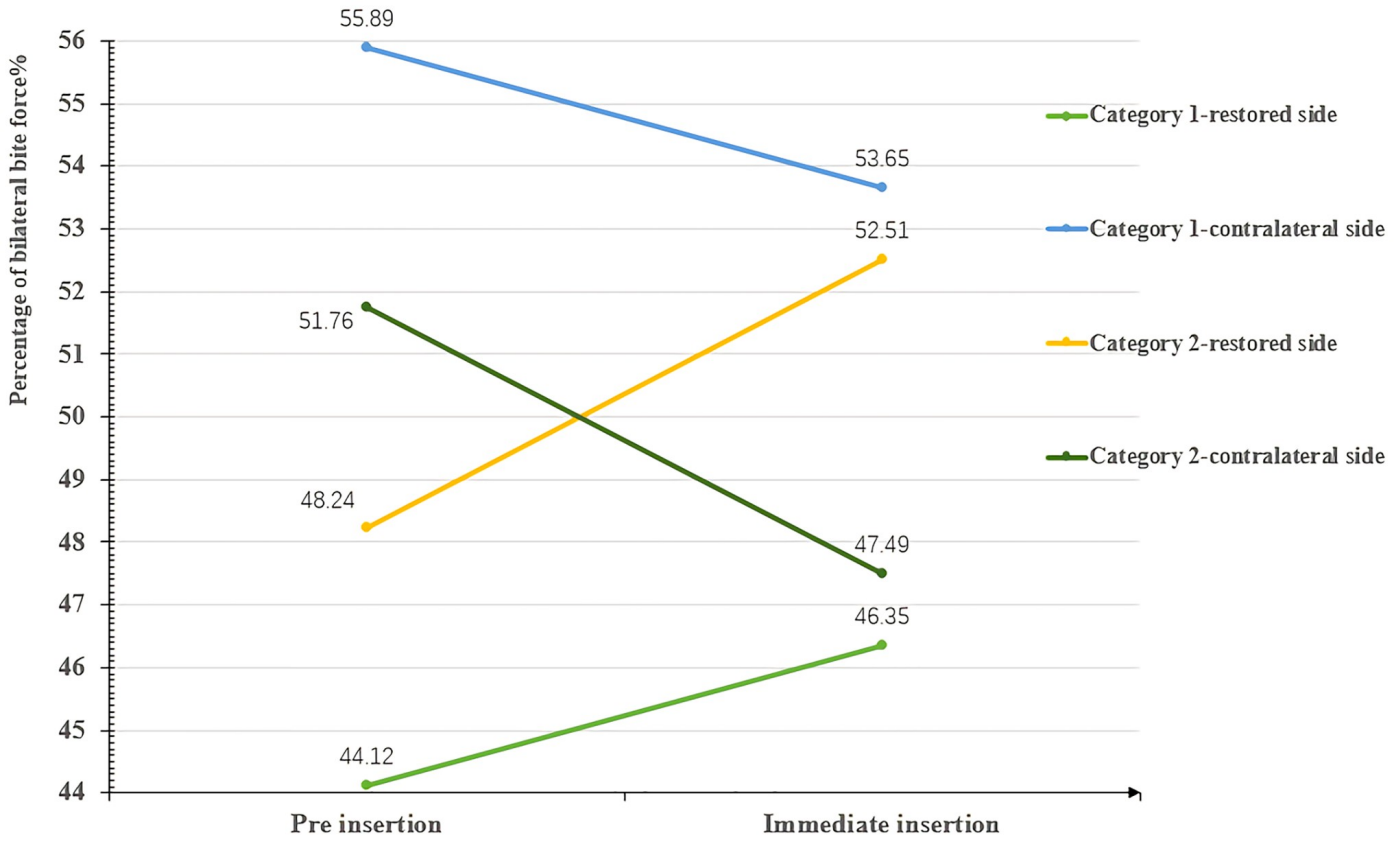
Changes in the average bilateral bite force (before treatment vs immediately following restoration).

**Fig 5:**
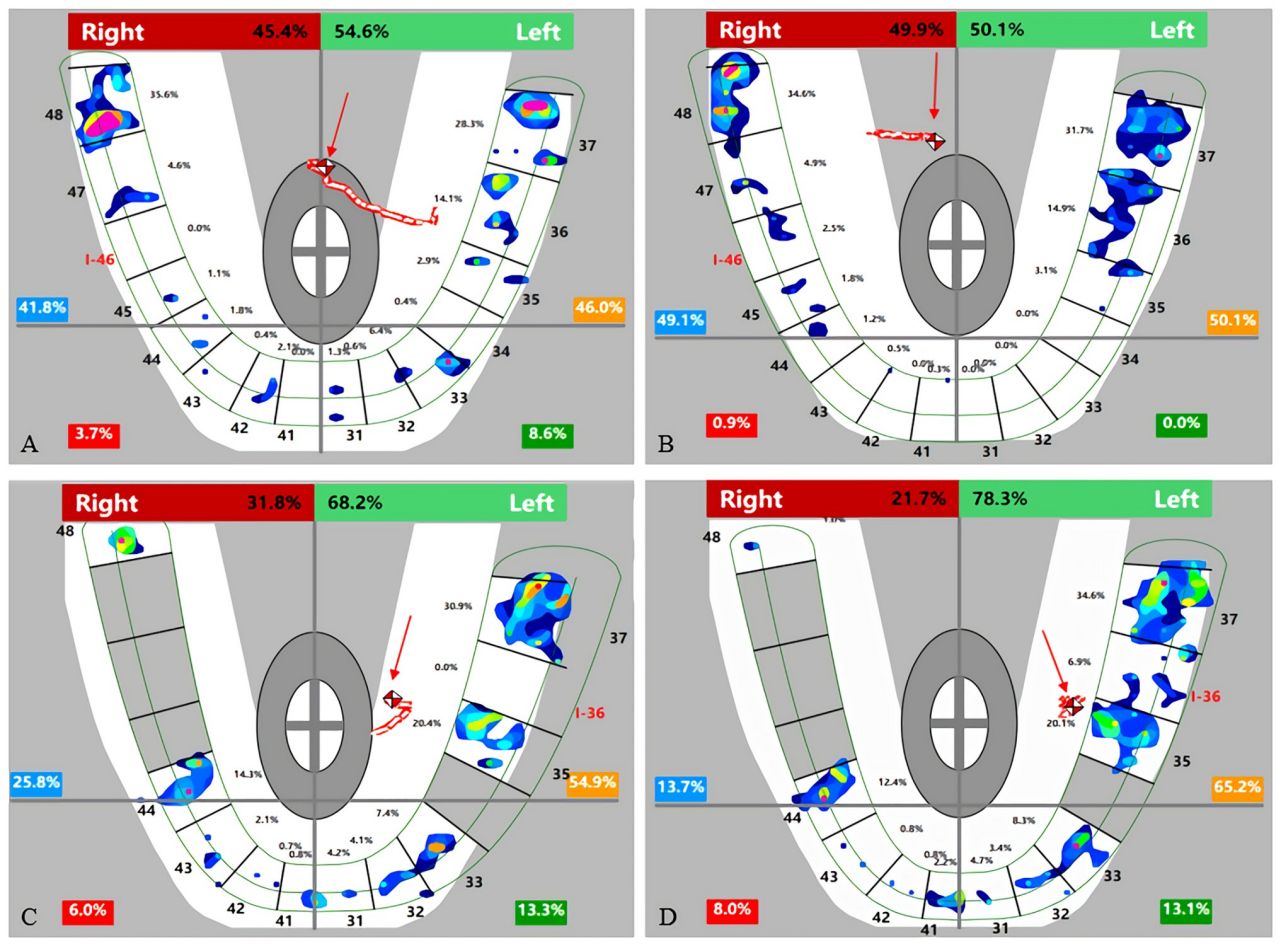
T-scan images of the occlusal force distribution before and after placement of restoration. Red arrows reflect the center of bite force (red and white diamond icon). (A) Unilateral one missing: pre- 46 implant restoration insertion; (B) Unilateral one missing: post- 46 implant restoration immediate insertion; (C) Bilateral missing: pre- 36 implant restoration insertion; (D) Bilateral missing: post- 36 implant restoration immediate insertion.

Regardless of unilateral (category 1) or bilateral (category 2) missing teeth, the occlusal force of the posterior segment on the restored side increased significantly (*P* = .013,.001, respectively) and decreased (though not significantly) in the contralateral posterior segment and anterior segments (Table 4). Although the occlusal force of the posterior quadrants on the restored side increased, the gap between bilateral occlusal forces was reduced for subjects with unilateral edentulousness but increased for those with bilateral edentulousness (Figs 4 and 5).

**Table 4:** Comparison of the bite force distribution on segments (before treatment vs immediately following restoration).

	Restored side		*P* value	Contralateral side		*P* value
	Category 1 (n = 14)	Category 2 (n = 13)		Category 1 (n = 14)	Category 2 (n = 13)	
**Anterior**						
Pre	7.94 ± 8.43 (4.425)	13.86 ± 10.02 (13.850)	0.104	9.74 ± 11.59 (6.750)	13.69±11.36 (7.770)	0.244
Immediate	6.86 ± 8.05 (3.020)	11.32 ± 9.26 (11.900)	0.126	8.92 ± 9.91 (6.050)	10.87±9.76 (8.930)	0.497
*P* value	0.875	0.221		0.600	0.917	
**Posterior**						
Pre	36.18 ± 13.96 (36.880)	34.37 ± 16.23 (35.700)	0.698	46.15 ± 13.06 (47.140)	38.08 ± 15.97 (32.670)	0.207
Immediate	39.49 ± 13.11 (41.535)	41.20 ± 15.37 (43.230)	0.884	44.73 ± 12.56 (41.615)	36.62 ± 17.01 (35.900)	0.286
*P* value	0.013*	0.001**		0.510	0.221	
**Half arch**						
Pre	44.12 ± 10.72 (46.510)	48.24 ± 11.83 (42.930)	0.497	55.89 ± 10.72 (53.540)	51.76 ± 11.83 (57.070)	0.497
Immediate	46.35 ± 10.10 (47.115)	52.51 ± 12.79 (46.850)	0.308	53.65 ± 10.10 (52.885)	47.49 ± 12.79 (53.150)	0.308
*P* value	0.140	0.039*		0.140	0.039*	

* *P* < 0.05;

** *P* < 0.01;

Descriptive data: mean ± standard deviation and (median).

### Changes in the bilateral bite force deviation and occlusal force center

**Fig 6:**
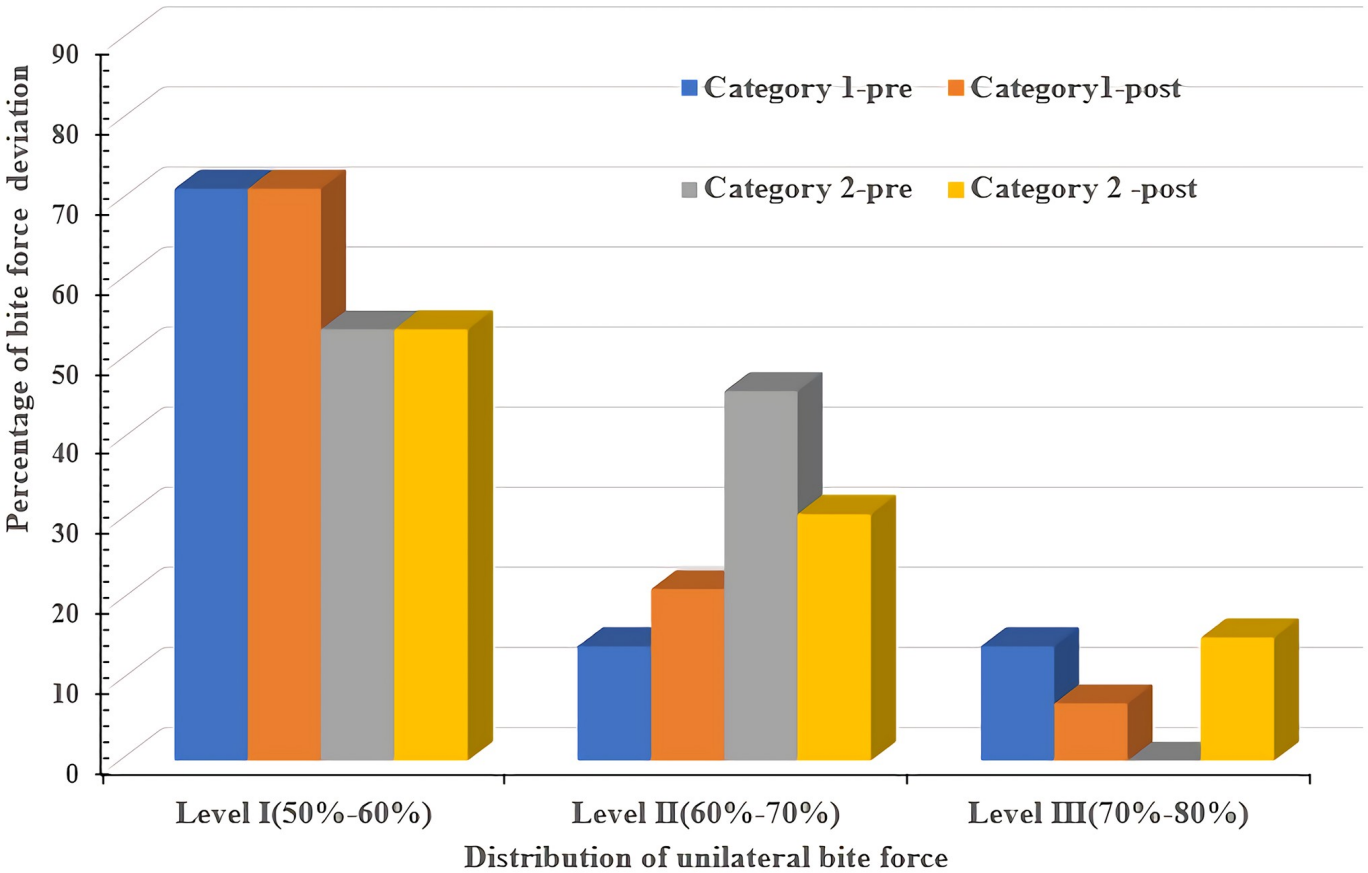
Changes in the bilateral force deviation (before treatment vs immediately following restoration).

The distribution of the bilateral bite force deviation levels was not significantly different pre- and post-treatment between the two categories. The level III bilateral force deviation was decreased postinsertion in unilateral defects, yet appeared in bilateral defects (Table 5 and Fig 6). Meanwhile, the results in Table 6 showed that there were significant differences in the initial COF position between types (FTU_R_ < FTU_C_) and (FTU_R_ *>* FTU_C_). The COF of type (FTU_R_ < FTU_C_) was more frequently located on the contralateral side, while that of type (FTU_R_ *>* FTU_C_) was more frequently located on the restored side. Regardless of the pre- or postplacement measurement, the COF of type (FTU_R_ = FTU_C_) was not statistically different from the above two types.

**Table 5:** Comparison of the bilateral bite force deviation (before treatment vs immediately following restoration).

		Category 1 (n = 14)	Category 2 (n = 13)	*P* value
**Pre**	Level I	10 (71.43%)	7 (53.85%)	
	Level II	2 (14.29%)	6 (46.15%)	
	Level III	2 (14.29%)	0	0.105
**Immediately**	Level I	10 (71.43%)	7 (53.85%)	
	Level II	3 (21.43%)	4 (30.77%)	
	Level III	1 (7.14)	2 (15.38%)	0.616
**P value**		0.766	0.301	

Note: Bite force distribution of one side: 50%-60% (level I); 60%-70% (level II); 70%-80% (level III).

**Table 6:** Comparison of the occlusal force center location of different functional teeth units (before treatment vs immediately following restoration).

	FTU_R_ <FTU_C_	FTU_R_ =FTU_C_	FTU_R_>FTU_C_	P value
**Pre**	(n = 16)	(n = 8)	(n = 3)	
Restored side	4 (25.00%)	2 (25.00%)	3 (100%)	
Contralateral side	12 (75.00%)	6 (75.00%)	0 (0%)	0.034*
**Post**	(n = 0)	(n = 16)	(n = 11)	
Restored side		4 (25.00%)	6 (54.55%)	
Contralateral side		12 (75.00%)	5 (45.45%)	0.224

Note:

*P < 0.05;

Functional tooth unit of Restored side (FTU_R_); Functional tooth unit of Contralateral side (FTU_C_); Pre: FTU_R_ < FTU_C_ vs FTU_R_ *>* FTU_C_ (*P* = 0.036 < 0.05).

### Changes in bite force distributed on implants and natural teeth with time

**Fig 7:**
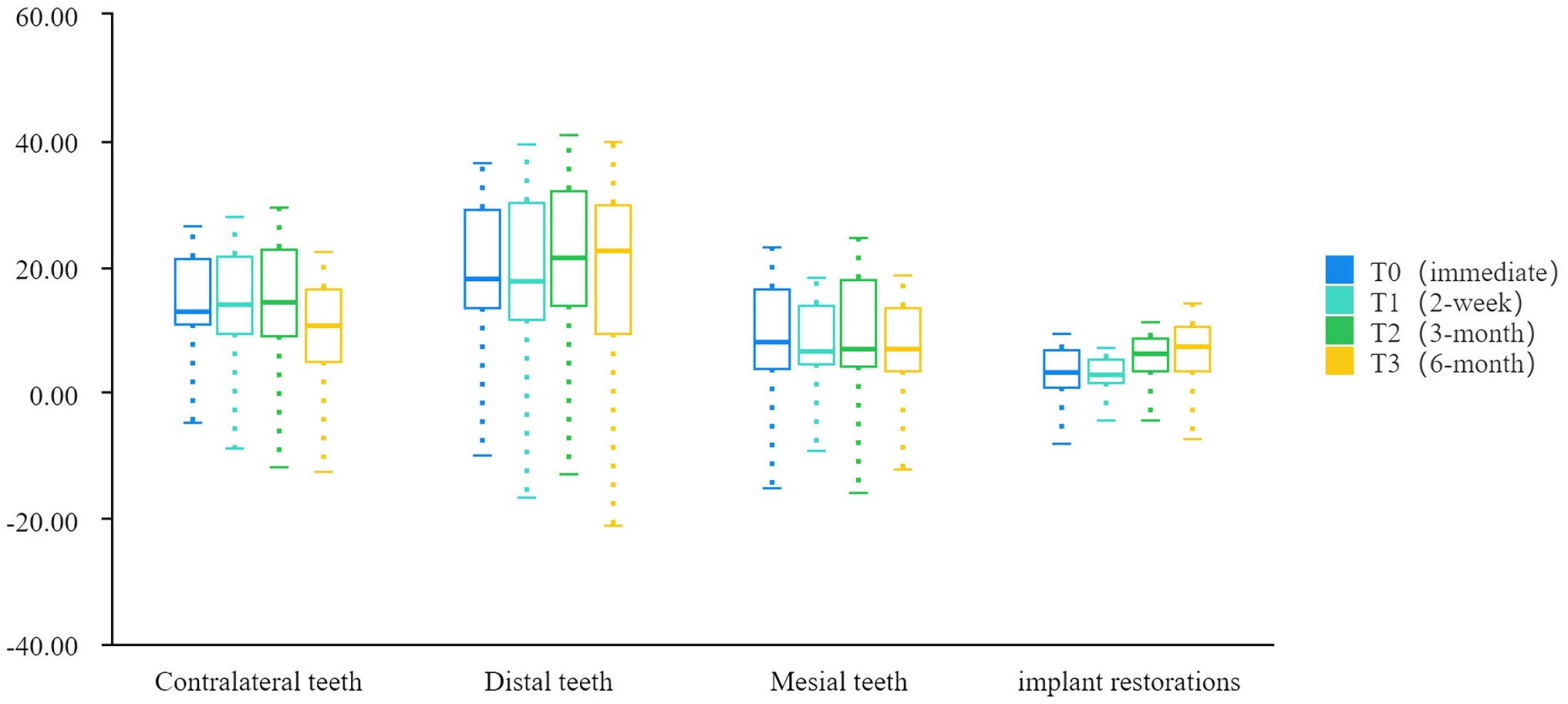
Comparison of the bite force distributed on implant restorations and teeth at different stages.

**Fig 8:**
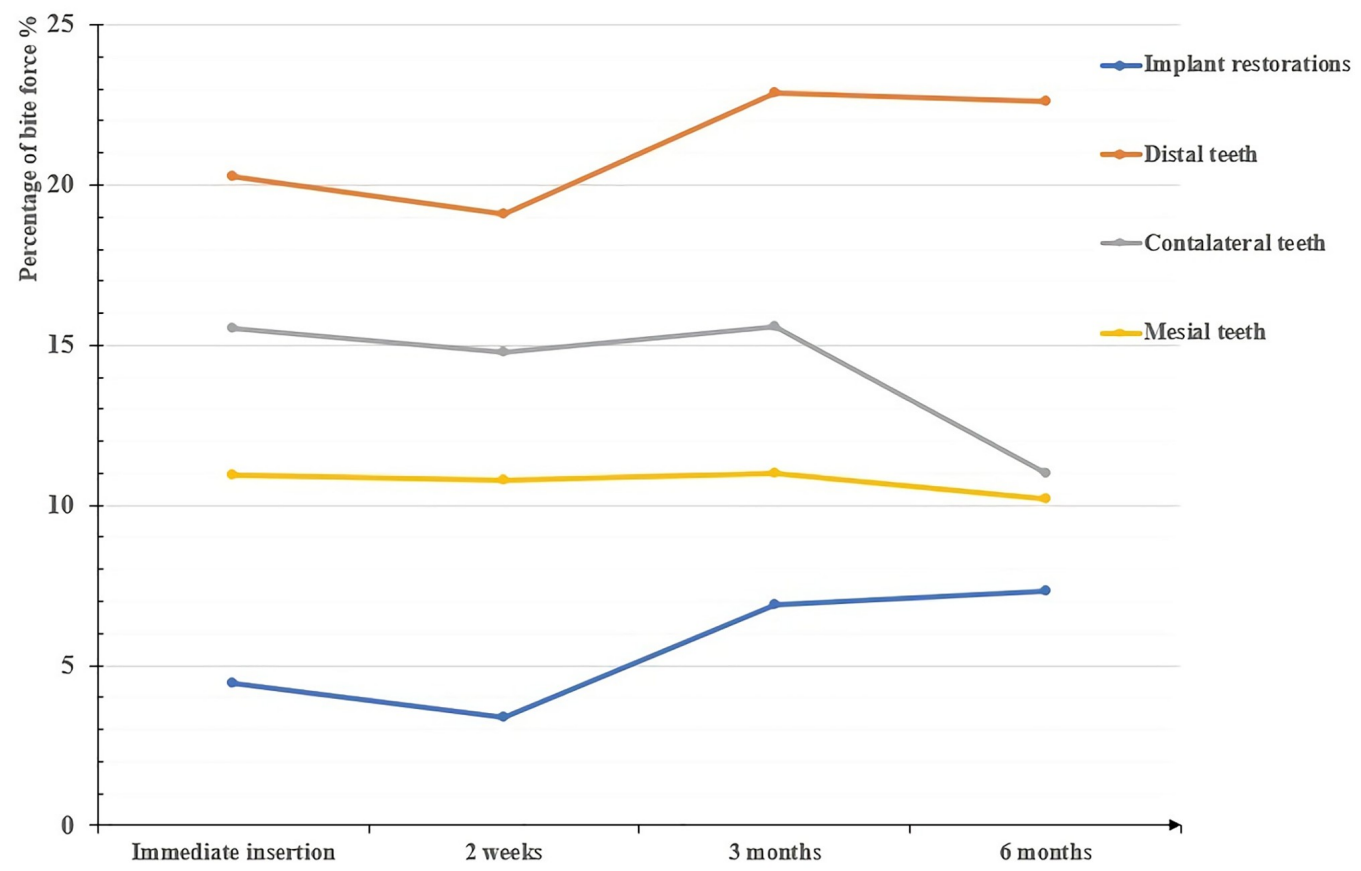
Changes in the average bite force distributed on the teeth and implant crowns over time.

**Fig 9:**
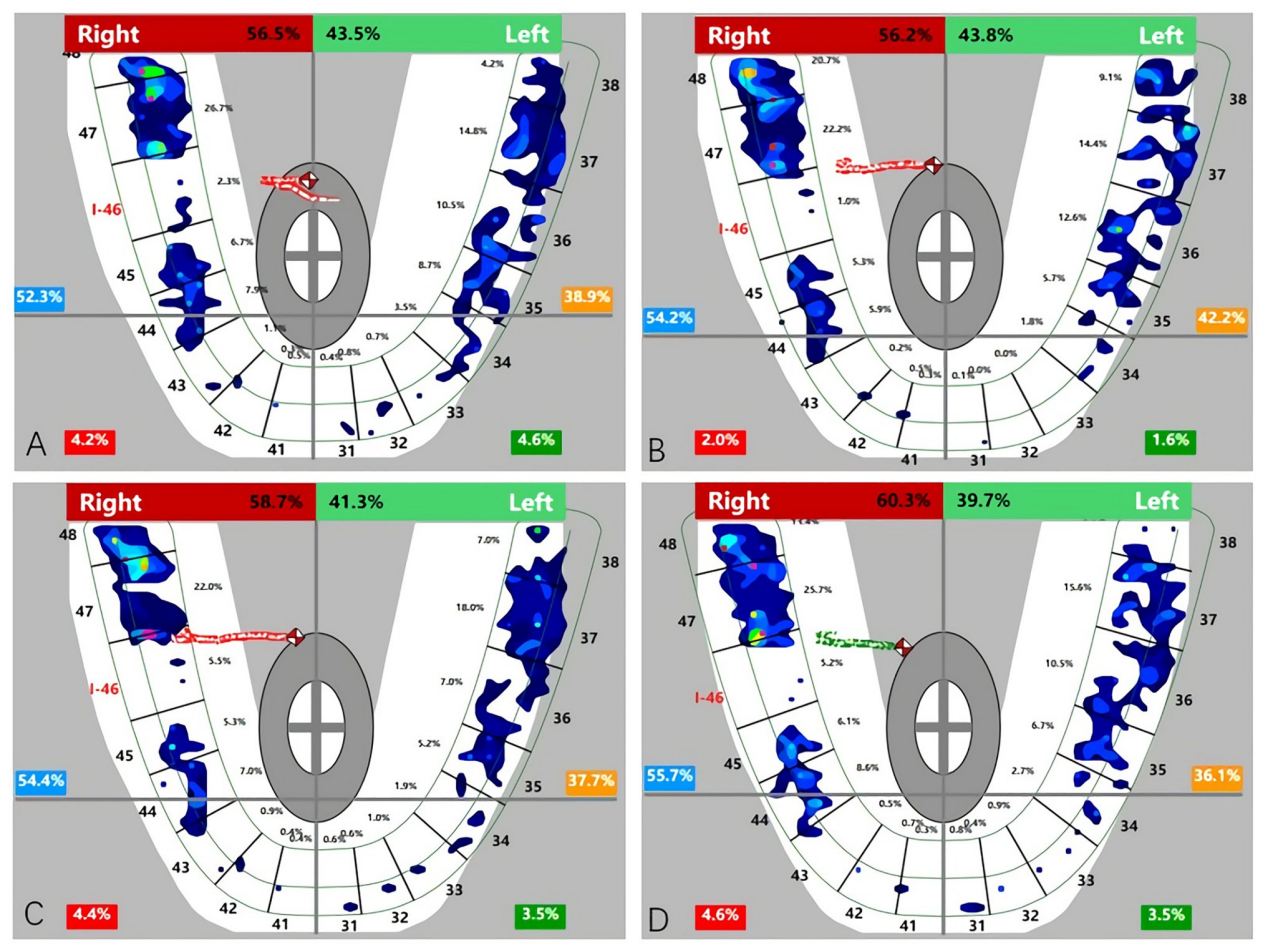
T-scan images of the bite force distribution on implant and teeth in different stages. (A) Post- 46 immediate insertion; (B) 2 weeks (final torque); (C) 3 months; (D) 6 months.

The bite force of the implant restorations was significantly lower than those of the control natural teeth initially, then it was not significantly lower than that of the mesial teeth at 3 months, and mesial and contralateral teeth at 6 months follow-up, respectively (Table 7, Fig 7). In addition, the average bite force distributed on implant restorations increased by 2.04 times after insertion and significantly (*P* = .008,.013, respectively) changed from 2 weeks to 3 months and 6 months post-insertion (Table 7, Figs 8 and 9).

**Table 7:** Changes in the bite force distributed on implants and natural teeth with time (Group 2).

	Implant	Mesial	Distal	Contralateral	*P* value
**Immediate(T0)**	n = 27	n = 27	n = 21	n = 19	
	4.46 ± 4.30 (3.270)	10.94 ± 11.27 (7.950)	20.27 ± 10.24 (18.070)	15.54 ± 9.99 (12.900)	0.000**
**2-week (T1)**	n = 17	n = 17	n = 14	n = 13	
	3.39 ± 2.61 (2.700)	10.79 ± 12.60 (6.300)	19.09 ± 9.46 (17.465)	14.80 ± 9.01 (13.830)	0.000**
**3-month (T2)**	n = 26	n = 26	n = 20	n = 18	
	6.90 ± 4.77 (6.280)	11.01 ± 11.72 (6.675)	22.86 ± 10.63 (21.500)	15.57 ± 8.88 (14.275)	0.000**
**6-month (T3)**	n = 18	n = 18	n = 14	n = 12	0.001**
	7.31 ± 4.60 (7.385)	10.20 ± 12.28 (6.800)	22.59 ± 14.65 (22.500)	10.98 ±6.11 (10.400)	
***P* value**	0.003**	0.277	0.871	0.028*	

Note:

*P < 0.05;

**P < 0.01;

Immediate: contralateral *>* implant (*P* = 0.000 < 0.01); distal *>* mesial (*P* = 0.002 < 0.01); distal *>* implant (*P* = 0.000 < 0.01); mesial *>* implant (*P* = 0.013 < 0.05); 2 weeks: contralateral *>* implant (*P* = 0.000 < 0.01); distal *>* implant (*P* = 0.000 < 0.01); distal *>* mesial (*P* = 0.006 < 0.01); mesial *>* implant (*P* = 0.027 < 0.05); 3 months: contralateral *>* mesial (*P* = 0.021 < 0.05); contralateral *>* implant (*P* = 0.001 < 0.01); distal *>* implant (*P* = 0.000 < 0.01); distal *>* mesial (*P* = 0.000 < 0.01); 6 months: distal *>* implant (*P* = 0.000 < 0.01); distal *>* mesial (*P* = 0.001 < 0.01); Bite force of Implant restoration: 3 months *>* 2 weeks (*P* = 0.008 < 0.01); 6 months *>* 2 weeks (*P* = 0.013 < 0.05).

**Fig 10:**
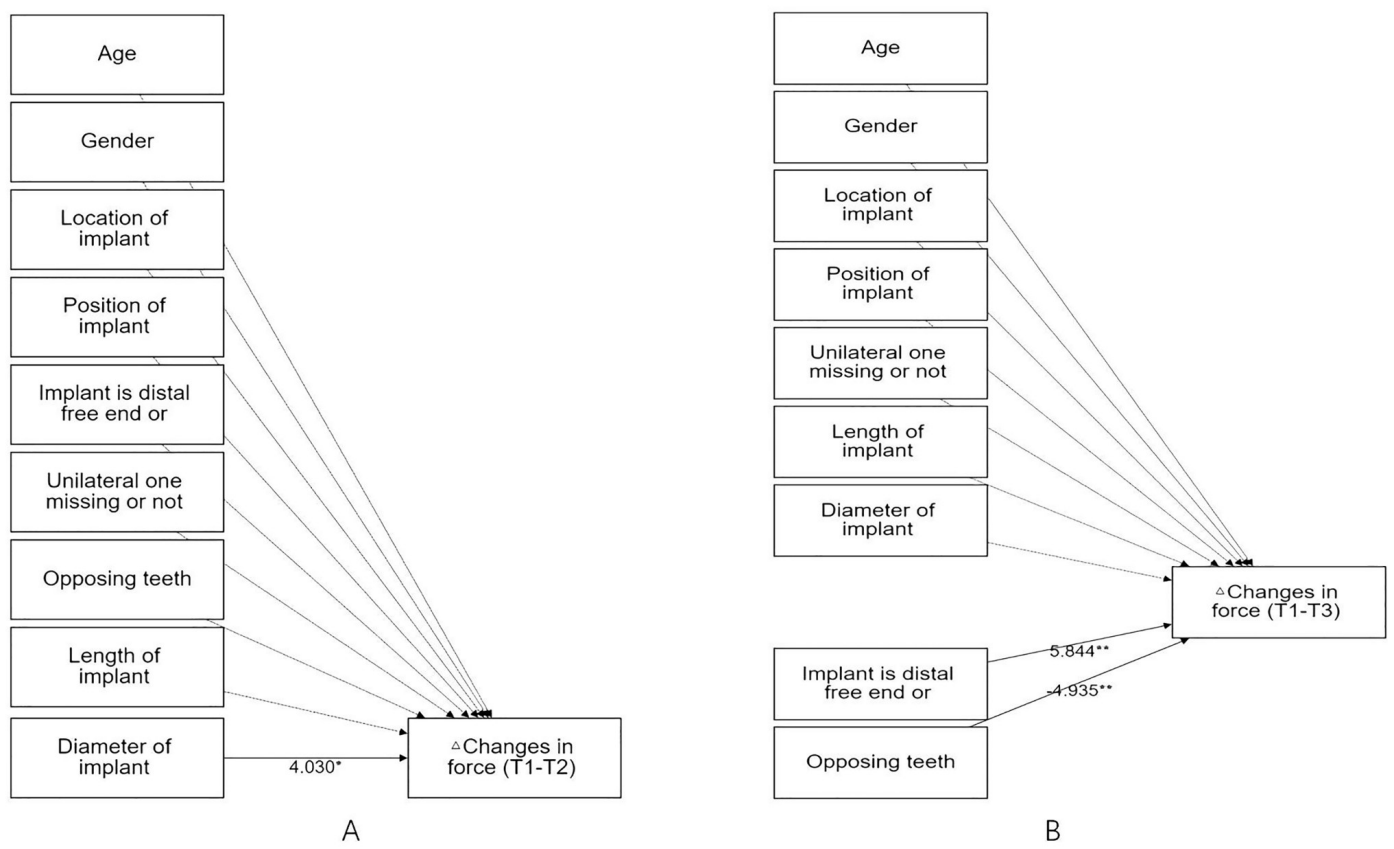
Diagram of the stepwise regression model of force changes at 3 and 6 months. (A)ΔChanges in force (T1-T2): the amounts of changes in bite force of implant between 2 weeks and 3 months; (B)ΔChanges in force (T1-T3): the amounts of changes in bite force of implant between 2 weeks and 6 months.

Analysis of stepwise regression at different stages showed that implant length (*P* = .012), sex (*P* = .027) and initial category of defect (*P* = .008), implant diameter (*P* = .044), implant is distal free end (*P* = .001) and opposing teeth (*P* = .002) had significant effects on the force distribution of implant restorations when measured immediately following insertion and 2 weeks and 3 months (ΔChanges in bite force from 2 weeks to 3 months) and 6 months (ΔChanges in bite force from 2 weeks to 6 months), respectively (Table 8 and Fig 10).

**Table 8:** Stepwise regression of the force distributed on the implant at different times (Group 2).

	Independent variable	Regression coefficient	95%CI	*P* value	VIF	R^2^	F
**Force of implant (T0) n = 27**	Length of implant	-1.560	-2.691~-0.429	0.012*	1.000	0.226	7.312
							*P* = 0.012*
**Force of implant (T1) n = 17**	Gender	-2.321	-4.161 ~ -0.482	0.027*	1.001	0.522	7.643
							*P* = 0.006**
	Unilateral one missing or not	-3.431	-5.595 ~ -1.267	0.008**	1.001		
**Force of implant (T2) n = 26**	Implant is distal free end or not	4.070	-0.060 ~ 8.201	0.065	1.000	0.135	3.730
							*P* = 0.065
**ΔChanges in force (T1-T2) n = 17**	Diameter of implant	4.030	0.446 ~ 7.613	0.044*	1.000	0.245	4.858
							*P* = 0.044*
**Force of implant (T3) n = 18**	Implant is distal free end or not	7.330	3.480~ 11.181	0.002**	1.000	0.465	13.920
							*P* = 0.002**
**ΔChanges in force (T1-T3) n = 13**	Implant is distal free end or not	5.844	3.409 ~ 8.279	0.001**	1.000	0.803	20.390
							*P* = 0.000**
	Opposing teeth	-4.935	-7.175 ~ -2.695	0.002**	1.000		

Note:

*P < 0.05,

**P < 0.01;

Immediate (T0), 2 weeks (T1), 3 months (T2); 6 months (T3);ΔChanges in force (T1-T2): the amounts of changes in bite force of implant between 2 weeks and 3 months; ΔChanges in force (T1-T3): the amounts of changes in bite force of implant between 2 weeks and 6 months

## Discussion

This study found that at least 3.31 units of occlusal force significantly increased (*P* < 0.05) in the posterior segment of the restored side after delivery of single posterior dental implant restoration, and the occlusal force of the implant crown significantly increased (*P* < 0.01) during the 3-month follow-up. Therefore, the null hypothesis that implantation of single posterior restorations could not bring about a significantly increased bite force in the posterior segment of the restored side when immediately following placement, and the occlusal force of implant restorations would not change significantly within a short period of 6 months was rejected.

Implants respond differently from natural teeth under loading [2, 4, 28]. The PDL of the natural tooth absorbs shock, yet the occlusal load is directed into the crestal bone in an implant prosthesis. Implants generate greater stresses and strain at the crest of bone than a natural tooth under similar loading conditions because the elastic modulus of the tooth is closest to that of bone [28]. The stress around the interface between the implant component and the bone implant exceeds what is biologically acceptable and is often regarded as a potential cause of peri-implant bone loss and failure of the implant/implant prosthesis [2, 8]. Unlike conventional detection methods,“light contact”was interpreted from the T-scan in terms of both the percentage of bite force and the density of bite force with different colors. On the one hand, this was an effective way to accurately grasp the changes and correlations of the occlusal force distributed on the implants and the natural teeth by comparing the relative occlusal force value of those teeth in view of the fact that“force distribution should be equal bilaterally and maximized on adjacent teeth”[1, 7]. On the other hand, the colored spots reflect different levels of bite force density instead of the size of the mark; a clinical adjustment is commonly made based on the size of the mark, but studies have found no scientific correlation between the articulating paper mark size and the amount of applied force [20–22]. In other words, the antagonistic tooth morphology is likely the key factor because small traces may result when a sharp surface opposes a flat surface, or contacts other pointy surfaces. Large variations in both the percentage and proportion of the contralateral tooth bite force were observed (Fig 2), and a high density of bite force was found (Fig 3). Besides initial occlusion, such differences may also caused by the operator’s experiences and adjustments may base on patient feedback such as supra-occlusion feeling.

The results of group 2 showed that the bite force of implant restorations was still significantly (*P* = .000) lower than that of the distal teeth during the 6-month follow-up (Table 7, Fig 7); however, the observed bite force of the implant increased significantly (*P* = .008,.013) from 2 weeks (3.39 ± 2.61%) to 3 months (6.90 ± 4.77%) and 6 months (7.31 ± 4.60%), respectively, while that of contralateral teeth decreased (*P* = .307) from immediately following restoration (15.54 ± 9.99%) to 6 months (10.98 ± 6.11%) (Fig 8). The average bite force between contralateral teeth and implant crowns decreased from 3.48 initially to 1.50 times and from 4.54 to 3.09 times for distal teeth and implant crowns over time (Table 7, Fig 8), respectively. The findings of this study were consistent with related research [9, 16, 29, 30], which presented bite force of prosthesis increased from 4.72% to 9.64% at 3 months [29] whereas there was no significant difference in bite force of prosthesis from 3 months (9.87 ± 6.79%) to 6 months (10.59 ± 6.59%) [9]. The continuous eruption of the opposing teeth and the occlusal wear of the remaining natural teeth [31] may partly explain the variation in the occlusion of implant prostheses. A clinical research revealed that a yearly wear rate of 29 μm for molars and 15 μm for premolars [32], and occlusal wear could modify the distribution of occlusal stresses and lead to a passive eruption of natural teeth [33]. Also, it has been reported that the load-bearing capacity of indirect restorations and long-term outcome were affected by the marginal and internal adaptation of the restoration [34–36], and a more complex occlusal geometry of implant prosthesis determined by the antagonist may harm internal fits, these may be the confounding factors of the changes in bite force distribution as well. The regression correlation of -4.935 (*P* = .002) revealed that the opposing teeth had a significant negative impact on the changes in occlusal force from 2 weeks to 6 months, this performance may be caused by the reasons all mentioned above. Moreover, the bone remodeling and improved osseointegration around the implant might play an important role. Bone tissue is characterized by a constant turnover in response to loading, and dynamically remodels over several years until more favorable alignment results in higher mechanical properties [37]. Implant restoration can withstand a greater bite force as the contact area between the implant increases and denser bone forms [38]. Furthermore, the occlusal adjustment and adaptation of implant loading is a complex feedback process that may involve changes in central nervous system plasticity. The sensorimotor central control system is characterized by neuroplasticity [39], resulting in the adaptive response of the corresponding area of the motor cortex to changes in the occlusal form and tooth status after implant restoration loading. Analysis of the stepwise regression showed that implant length had negative effects on the distribution of the occlusal force on implant restorations immediately following insertion. In fact, the baseline data were affected by the doctor’s experience and individual occlusal characteristics to a large extent. The Spearman test result of -0.276 indicated that there was no significant correlation (*P =* .163) between the implant length and the value of bite force distribution, so the influencing factor at that stage was probably the confounding contributions of multiple factors mixed, which were insufficient to prove that implant length was the key factor. At 2 weeks, a routine occlusal adjustment was performed again after the final torque was used. Most importantly, after measurement immediately following restoration, adjustments were made to the unilateral defects in patients that were determined to be high-density to reduce the bite force in case these were the main causes of the decreases in bite force for subjects with unilateral defects at 2 weeks. From 2 weeks to 3 months, the occlusal force of the implant significantly increased. Therefore, the amounts of the changes in bite force from 2 weeks to 3 months were included as dependent variables. The model fitting revealed that 24.5% of the changes in bite force could be explained by implant diameter, and there was a significantly positive regression correlation of 4.030 (*P* = .044) between the two (Table 8, Fig 10). The Spearman result showed a significant correlation of 0.482 (*P* = .050) between the diameter and change in bite force. This result supported that the increased bite force of the implant resulted from the continuous improvement of bone remodeling and osseointegration around the implant. It should be pointed out that 13 of the 17 subjects compared at the 3-month follow-up had the same implant length (10.0 mm), regarding diameter, a relatively wider implant is beneficial for more osseointegration since its increases the implant surface area and hence bone-to-implant contact [40, 41]. Moreover, it has been reported that the use of wider implants may better dissipate the acting forces and thus reduce the stress in the cortical bone surrounding the implant [42]. Hence, implant restoration with wider diameters may result in a better bite force distribution under the adaptive adjustment of the sensorimotor central control system. Besides, the study of occlusion is extremely complex, it should be noted that the new implant-supported crown had been placed in 5 patients and 2 patients had replaced the new crown of a natural tooth when the 6-month follow-up, these may contribute to the changes in bite force distribution of the implant restorations. The result of 6 months showed that posterior implant restorations in distal free end bear relatively higher bite forces, which was similar to restorations positioned more posteriorly leading to an increase in pressure [27], this was in line with the occlusal environment determined by physiological curvature of the jaw and condylar morphology.

The results of the measurement immediately after insertion indicated that the occlusal force of the posterior segment on restored side significantly increased by 3.31% or 6.83% after placement of a single posterior implant restoration. This result was supported by a study on the redistribution of occlusal force after insertion of a single posterior dental implant restoration [27], which reported a 4.18% increased bite force for subjects missing a single tooth. Specifically, when this chewing unit was inserted into the unilateral missing side or one side of the bilateral missing, a decreased occlusal force of the contralateral posterior segments and anterior teeth was found with a significant increase in the restored posterior segments. It is well known that fragmentation of food depends on the occlusal area of the teeth, which is primarily determined by the number of functional teeth [43]. Similarly, as an important indicator for evaluating the functional status of the chewing system, maximum bite force decreases are associated with compromised dentitions, such as loss of teeth and occlusal contacts [44]. The addition of an FTU leads to increased contact points and surface area, which causes the pressure to be redistributed. It can be inferred that the improvement of chewing performance is accompanied by an increased bite force and mastication units on the restored side since there was a correlation between bite force and chewing ability [45]; the bite force and number of occlusal units are also key to masticatory performance [45, 46]. In a study of patients who received one single, two single and three single implant-supported fixed restoration treatments, the number of restorative units was thought to be a more important factor in explaining the improvement in both mastication performance and patient satisfaction in their chewing ability on the treatment side [47].

Although similarly increased bite forces were found, the discrepancy in the bilateral force distribution, including the deviation and location of the occlusal force center between unilateral defects (category 1) and bilateral defects (category 2), should not be ignored. First, the average bilateral bite force gap of unilateral defects (category 1) was reduced from the initial 11.77% to 7.3%, while it was increased from the initial 3.52% to 5.02% of bilateral defects (category 2) (Table 4 and Fig 4). Second, when comparing the levels of the bilateral force deviation [48] of 2 categories, the proportion of the level II deviation increased by 7.14% and that of the level III deviation decreased by 7.14% in the first category, yet the proportion of the level III deviation increased by 15.38% and that of the level II deviation decreased by 15.38% in the second category (Table 5, Fig 6). These results can be explained by the initial occlusion and mechanism of the center of bite force. The COF marker pinpoints the location of the sum of the total force of the occlusal contacts, which is done by calculating the sum of the mediolateral and anteroposterior force moments of the recorded contacts and presenting these data by superimposing the COF marker on the tooth contact data [49, 50]. This process serves as a guide for the dentist to compare the patient’s occlusion to that of a group of normal subjects. After a single posterior implant restoration was inserted, the increased posterior contacts were conducive to obtaining more occlusal force distribution, which resulted in the COF moving towards the inserted side (Fig 5). This trend manifested as a reduction in the bilateral deviation for category 1 subjects because the contralateral side was the dominant side before insertion (Table 6). In contrast, only 2 subjects (15.38%) in category 2 had fewer FTUs on restored side before insertion; for the other 11 subjects (84.62%), the occlusal force and contacts of the inserted side were not in a weak position before insertion, and 3 of them showed absolute dominance on the restored side (Table 6). Therefore, the added functional occlusal unit on the restored side amplified the effect, which likely strengthened the strong side but weakened the weak side (Fig 5). This trend presented as a widening bilateral gap (Table 4, Fig 4) and increased bilateral force deviation (Table 5, Fig 6). In an ideal occlusion, the bite force is evenly and symmetrically distributed on the left and right sides of the dental arch, and a minimized bilateral bite force gap is advocated for the balance of occlusion since a severely unbalanced bite force distribution originates from asymmetrical occlusal contact patterns, which is not only unfavorable for occlusion stability but also possibly causes pathological hazards [51, 52]. From this perspective, the effect of reduced or increased bilateral bite force gaps played a positive or negative role in promoting the balance of occlusion, respectively. Therefore, we suggest that single implant restoration should be placed prior to the obvious weaker side of occlusal contacts for patients with bilateral edentulousness.

The limitations of this study include the potential bias from the small sample size and short-term follow-ups. The dropout rate was 15.63% (5 lost from 32 subjects) at 3 months and 18.75% (6 dropout from 32 subjects) at 6 months, respectively, so it was merely a pilot study. Since T-scans use percentages rather than absolute force values and comprehensive treatment plans and long-term outcomes are affected by multiple factors, randomized clinical studies with more samples are needed.

## Conclusions

With the limitations of this study, the following conclusions were drawn:

- An improvement of bite force and masticatory ability can be achieved with the delivery of a single posterior implant restoration, which was mainly reflected in the significantly increased bite force on the restored posterior segment.
- During the four-phase postinsertion observation period, the occlusal force of single posterior implant restorations increased, yet it was still significantly less than that of distal teeth at 6- month follow-up, prompting us to determine the periodontal status and bone loss rather than the value of bite force alone; although, bite force is meaningful, a regular follow-up and occlusion assessment are strongly needed.

## Supporting information

S1 File Research protocol. (PDF)

S2 File Encoded data for analysis. (XLSX)

S1 Checklist TREND statement checklist. (PDF)
