# Supplementary material for: Digital occlusal analysis of pre and post single posterior implant restoration delivery: A pilot study
Source: PLoS One. 2021 Jul 2;16(7):e0252191. doi: 10.1371/journal.pone.0252191 (PMC8253389; doi:10.1371/journal.pone.0252191)
Supplement: S1 File — (PDF) [file pone.0252191.s001.pdf]

Research Protocol

Occlusal analysis of pre and post crown insertion of posterior single-implant restoration

BY

Mrs ZHOU TING

FACULTY OF DENTISTRY
THAMMASAT UNIVERSITY
ACDEMIC YEAR 2019

Research Protocol

The Ethical Review Sub-Committee Board for

Human Research Involving Sciences, Thammasat University, No. 3

****

1. Cover
2. Research Protocol name in Thai

การวิเคราะห์การสบฟันก่อนและหลังการบูรณะด้วยครอบฟันบนรากฟันเทียมในฟันหลังซี่เดียว

Research Protocol name in English …………Occlusal analysis of pre and post crown insertion of posterior single-implant restoration

3. Principal Investigators/Co-Investigators including percentage of workloads

3.1 Principal investigator (Zhou Ting)

Responsible for completing occlusion measurements and data collection, the workload accounts for 85%.

3.2 Co-Investigators(Tortrakul Pornsuksawang, Pichaya Mekcha and Tuangkun Mukkayadachochai)

Mainly assisting the principal investigator in explanation to the research subjects when necessary, and contacting the patients for follow up, the workload accounts for 15%.

4. Background and Signification of the research problems

Dental implants have been extensively used for oral reconstruction of partial and complete edentulism [1]. They have high survival and success rates, however, they are not immune to complications [2,3]. It is important to identify factors that can play a role in the initiation and the progression of peri-implant condition deterioration [4]. Occlusal overload is often regarded as the main causes for peri-implant bone loss and implant/ implant prosthesis failure [5-7]. Compared with natural dentition which supported by periodontal ligament(PDL),the mean values of axial displacement of teeth in the socket are 25–100 μm vertically and 56-108 μm buccolingually, whereas the range of motion of osseointegrated dental implants has been reported approximately 3–5 μm vertically and 10-50 μm laterally [8-10]. Additionally, dental implants exhibit low tactile sensitivity and low proprioceptive motion feedback because of the absence of periodontal mechanoreceptors[11].Under loading, the compressibility and deformability of periodontal ligament (PDL) in natural teeth can make differences in force adaptation compared with osseointegrated implants[9]. Implant generates greater stresses and strain at the crest of bone than a natural tooth which has similar loading conditions because of the elastic

modulus of the tooth is closest to bone [12]. It is therefore believed that dental implants may be more prone to occlusal overloading which refers to stress around the implant components and bone implant interface that is not both technically and biologically acceptable, which is often regarded as one of the potential causes for peri-implant bone loss and failure of the implant/implant prosthesis[1].

Dental occlusion plays a central role in clinical dentistry and is essential for normal physiologic function [13].It means the contact relationship between the upper and lower teeth, and also refers to the contact action or process between the upper and lower dentition. Occlusion can be defined as “static” teeth contact when the mandible is at rest relative to the maxilla, or "dynamic" teeth contact as the mandible moving relative to the maxilla [14]. For a single implant prothesis, the recommendations of occlusion are as follows: the force distribution should be equal bilaterally and maximized on adjacent teeth [15,16]. Light contacts at heavy bite and no contact at light bite in maximum intercuspation position(MIP) are considered a reasonable approach to distribute the occlusal force on teeth and implants[17].Light to medium occlusal contact in maximum intercuspation is recommended for the adjacent, natural teeth, with lighter contact or clearance between the occlusal face and opposing tooth [17,18]. Anterior guidance is recommended in lateral and protrusive excursions. In lateral excursions, posterior teeth should avoid heavy forces in the lateral direction by discluding [10]. Avoiding Premature contacts[9] both working and nonworking contacts on implant restorations is vital to reduce shear forces in a nonaxial direction [16,19]. An important reason of these considers is overloading resulting from improper occlusion[20]. Implant occlusion should be examined not only in terms of conventional occlusal schemes but also from the standpoint of the role of overloading factors[1]. Despite the above guidelines have been extensive applied in clinical practice, some problems have been in the state of exploration, which including the range of overload that biologically acceptable is also unknown[21-23], the effect of single implant-supported restorations on occlusal pressure distribution remains questionable[24], and the stability of this new occlusion constructed by implant insertion is still in doubt[25]. It may result in the occurrence of potential occlusal overloading in the process of implant restoration, due to the habitual occlusion of the patient, the discrepancy of mobility between implant and natural tooth, the movement of natural teeth. Therefore, it is critical to analyze and re-evaluate the occlusion with post-insertion posterior single-implant restoration. Meanwhile, Force distribution between implants and natural teeth in a partially edentulous region can be accomplished with serial and gradient occlusal adjustments[17].

In the past, many occlusal studies have relied heavily on articulating papers, foils, and photocclusion to conduct analysis. However, articulating paper mark size has been shown to be an unreliable indicator of occlusal force[26,27] .None of the conventional methods, such as the use of articulating paper, shim-stock foil, and impression waxes were able to quantify occlusal contacts. Studies found no scientific correlation between the articulating paper mark size and the amount of applied force, which demonstrates the inadequacy of articulating paper in interpreting the occlusal load[28]. T-Scan is a digital occlusion analysis system that records and measures tooth contact, force, and timing in real-time using a thin, flexible, pressure-sensitive bite transducer embedded in a dental arch-shaped recording sensor[29-31]. Not only it can reflect the position of the occlusal contact point, and the area of the contact point, the strength and distribution of the contact point objectively, but also recording the instantaneous occlusal contact in the chewing cycle to the nearest 0.01 second dynamically, It is better than other occlusion recording methods on accuracy and sensitivity, That providing a good means of research for the quantitative study of occlusal balance. T-Scan is mainly composed of sensor, connection handle, computer and analysis software. The sensor is a thickness of 60-100 μm and placed on the occlusal surface of the subject. The diaphragm has a horseshoe-shaped sensing area with small wires arranged in a longitudinal and transverse direction. When the wire is subjected to the occlusion pressure, the current change which can be transmitted to the computer through the control device and analyzed by software occurs. After that, it is possible to detect the number of occlusal contact points and the change of the occlusal force in different parts with time. The T-scan III System[32] (Tekscan III, South Boston, MA) is the newest generation of occlusal Analysis applying in clinical and scientific research. Compared with the previous two generations of occlusion analysis systems, the T-scan III occlusion analysis system has a medium, lateral, anterior and habitual occlusion mode to create a personalized arch image. The bow image can check and analyze the occlusal contact from two-dimensional and three-dimensional perspective, detect the change of the occlusal contact synchronously with the change of time, and observe the occlusal contact distribution, position, contact intensity at each time point in the dynamic occlusion process. Furthermore, observing the distribution of bite force in different regions, the characteristics of bite force changing, the time of occlusion contact. It is a quantitative, objective and reliable "dynamic" occlusal evaluation method [33,34]. Some scholars[35-37] have used it for the analysis of implants occlusion which including

morphology and density, for the relationship between excessive force and bone loss, influence of occlusal loading on peri-implant clinical parameters and so on.

In summary, it is implied that heavy occlusal force and undesirable distribution of occlusal contacts may be factors of overloading, thus possibly leading to higher susceptibility to implant bone loss, implant fractures/loss, and prosthesis failures. Roque, M.A.'s study [24] showed that post-insertion of posterior implants cause significant changes in the bilateral bite force distribution. A research from Madani.A.S et al [25] concluded that intensity of occlusal contacts of implant supported prostheses opposed by natural dentition gradually increased after prosthesis insertion. In addition, it was reported that in 46% of patients, the occlusal load applied to implant prostheses changed within 18 months after prosthesis placement, and almost one-third of these changes took place during the first 6 months[38]. Based on the above considerations, this study intends to further analyze and evaluate the occlusal parameters including occlusion time(OT), disclusion time(DT), distribution of force, occlusion interference(OI) after insertion of posterior single implant, and regularly assess the occlusion changes to eliminate possible risk factors to achieve balanced occlusion and stability.

5. Research questions

5.1 What are the effects of single posterior tooth on the bite force distribution and occlusal time after insertion?

5.2 Whether the occlusal parameters including time, distribution, interference and so on will change after the insertion of single posterior implant?

5.3 How does this new occlusion created by the insertion of single posterior tooth change over the next period of time?

5.4 What factors do contribute to the establishment of balanced and stable occlusion with single posterior implant?

6. Objectives of Research

6.1 Using T-scan that is a digital occlusion recording method with high accuracy and sensitivity, to obtain dynamic occlusion characteristics of posterior single-implant restoration, which including initial contact point, occlusion and disclusion time, force distribution and force outlier, interference in the movement of mandible.

6.2 At the same time, analyzing and regularly re-evaluating of those to remove potential occlusal risky factors and maintain balanced and stable occlusion.

7. Hypotheses

Ho: There is no difference in occlusal parameters after posterior single-implant restoration insertion with the use of T-scan measurement.

Ha: There are differences in occlusal parameters after posterior single-implant restoration insertion with the use of T-scan measurement.

7.1 Bite force percentage (BFP)with single posterior implant restoration.

Ho: There is no significant difference in bite force percentage for single posterior implant restoration during the 3 and 6- month period with the use of T-scan measurement.

Ha: There are significant differences in bite force percentage for single posterior implant restoration during the 3 and 6 -month follow up period.

7.2 Distribution of bite force (DBF) between anterior and posterior/bilateral teeth

Ho: There is no significant difference in distribution of bite force between anterior and posterior/ bilateral teeth after posterior single-implant restoration insertion with the use of T-scan measurement.

Ha: There are significant differences in distribution of bite force between anterior and posterior/ bilateral teeth after posterior single-implant restoration insertion with the use of T-scan measurement.

7.3 Initial tooth contact (ITC)

Ho: There is no significant difference in the frequency of initial tooth contact of single implant restoration after posterior single-implant restoration insertion.

Ha: There are significant differences in the frequency of initial tooth contact of single implant restoration after insertion and 3 to 6-month follow up time.

7.4 Occlusion time (OT)

Ho: There is no significant difference in the occlusion time of MIP after posterior single-implant restoration insertion with the use of T-scan measurement.

Ha: There are significant differences in the occlusion time in MIP after posterior single-implant restoration insertion with the use of T-scan measurement.

7.5 Disclusion time (DT)

Ho: There is no significant difference in the disclusion time during the functional excursions after posterior single-implant restoration insertion with the use of T-scan measurement.

Ha: There are significant differences in the disclusion time during the functional excursions after posterior single-implant restoration insertion with the use of T-scan measurement.

7.6 Occlusion interference (OI)

Ho: There is no significant difference in occurrence of occlusion interference during lateral and protrusive movement after posterior single-implant restoration insertion with the use of T-scan measurement.

Ha: There are significant differences in occurrence of occlusion interference during lateral and protrusive movement after posterior single-implant restoration insertion with the use of T-scan measurement.

8.Conceptual Framework

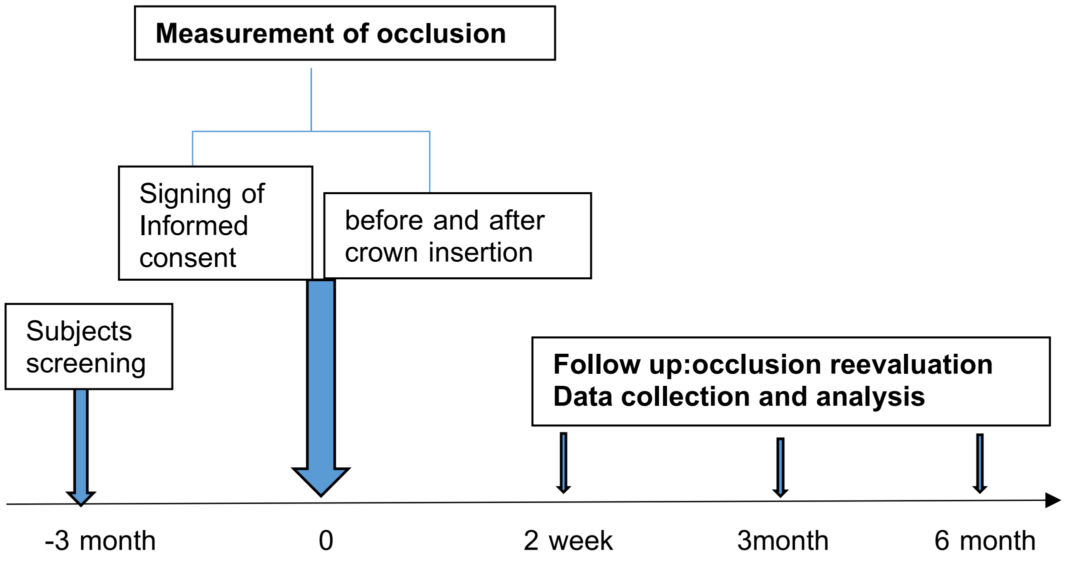

9.Scope of Study

The occlusal parameters of subjects will be evaluate at five phases of occlusion including before crown insertion, immediately after insertion and 2 weeks later,3 months and 6 months follow up period. 27 subjects who will be placed posterior single-implant restoration at Faculty of Dentistry, Thammasat hospital will be enrolled in this study.

10.Research Methodology

10.1 Study Population

27 subjects who will be placed posterior single-implant restoration at Faculty of Dentistry, Thammasat hospital will be enrolled in this study.

10.1.1 Inclusion criteria:

(1) Age:18-65 years old

(2) Single posterior dental implant, which will receive superstructure.

(3) Successfully osseointegration has been completed in the implanting area.

(4) In occlusion with natural dentition, the opposed teeth are natural teeth.

(5) There is only one posterior tooth missing space in the dentition or even if more than one missing space, the remaining edentulous region had no effect on the results during the study period.

(6) Absence of uncontrolled or untreated periodontal disease.

10.1.2 Exclusion criteria:

(1) severe temporomandibular disorders.

(2) use of known drugs that would affect the central nervous system or severe systemic diseases or known mental disorders.

(3) anterior open occlusion .

(4) history of bruxism.

(5) unwilling to accept further follow-up.

10.1.3 Sample Size Calculation

10.1.3.1 Reference variables

Reference variables from Article 1(Roque, M.A et al, 2017)and Article 2 (Madani, A.S., et al.,2017)

10.1.3.2 Information provided by reference materials

(1)Article 1: Prospective laboratory studies, no sample calculation source, only listed inclusion and exclusion conditions, using nonparametric statistical tests. Twenty-five cases were collected and four were lost to follow-up. Among the remaining 21, 10 are women and 11 are men.

(2)Article 2: Retrospective cohort study, self-controlled study design, no sample calculation source, only listed inclusion and exclusion conditions. And the full text does not give the final sample number, only the percentage, no absolute value.

10.1.3.3 Calculation method

(1)This study belongs to experimental research (not investigation), so the sample size is calculated according to the experimental research method (the proposed paper should specify the inclusion and exclusion criteria) . If the study is a prospective study, the study design of the repeated measurement data before and after insertion is based

on a hypothesis that the rate of loss of follow-up is 10%, and the analysis index is “The change of the bite force distribution with a single posterior implant restoration before and after insertion”, assuming that the bite force is the measurement data, the formula for calculating the sample size is as follows:

$$n=\left[\frac{(Z_{\alpha/2}+Z_{\beta})\ S}{\delta}\right]^2$$

(2)It is considered that the average increase of 2 units of the bite force before and after insertion can be considered significant. If the standard deviation of the difference of the bite force for the subjects in the study is σ=3 units, α is 0.05 on both sides, and the test efficiency 1-β is 0.90. Then the standard deviation of sample bite force s = (σ =) 3 units, δ = 2 units of bite force change, α = 0.05, $Z_{0.05/2}$ = 1.96, β = 0.10, $Z_{0.10}$ = 1.282 , which were brought into the formula to get 23.65, that is, at least 24 subjects need to conduct their own before and after experiments comparison. Taking into account 10% of the loss of follow-up, the final sample size is 24 × 110% =26.4, So total 27 subjects will be included in this study.

10.2 Materials and Equipments

10.2.1 Case Record Forms

Standard case record form (Appendix A) of the Implantology clinic, Faculty of Dentistry, Thammasat University, including:

10.2.1.1 General information

Patient’s demographic information. (e.g.name, age, gender, race, occupation, address, telephone number)

10.2.1.2 Medical record

Past and present medical history and treatment, medication outcomes.

10.2.1.3 Dental record

Chief complaint, dental history, clinical and radiographic examinations, procedures and outcomes.

10.2.1.4 Clinical examinations

(1) Extra-oral examination: general appraisal, skull, facial form, skin, eyes, neurologic deficit, lymph nodes of head and neck, TMJ, masticatory muscles, opening pattern, range of motion, salivary glands, muscles of head and neck

(2) Intra-oral examination: lip, buccal mucosa, salivary flow rate, oropharynx, soft palate, hard palate, tongue, floor of mouth

(3) Periodontal status

(a)Gingiva: gingiva biotype, gingiva recession, gingiva IDP, frenum attachment

(b) Local factors of Periodontal disease: local factors, risk factors, occlusal trauma, nutrition, parafunction habits, diagnosis of periodontal disease, prognosis, treatment plan

(4)Dental implant examination: Radiographic finding, Operation, Bone augmentations, Implant location

(5) Canine relationship

(6) Anterior relationship

(7) Midline

(8) Space condition

(9) Treatment record

10.2.2 Main Materials and equipment

(1)T-scan III bite analyzer

(Software version 9.0.1, Tekscan, Inc., Boston, MA, USA)

(2)T-scan Sensor (Tekscan, Inc., Boston, MA, USA)

(3) Computer

10.3 Ethical Approval

This clinical study protocol will be submitted for Ethical approval of the Ethics Committee in Human Research of Thammasat University prior to study initiation.

10.3.1 Access to complete medical records

In addition to complete cases recording, relevant imaging data (Panoramic, 3D CT) , as well as models and photographs of patient are all required.

10.3.2 Signing of informed consent

Once subjects will be fulfilled the inclusion/exclusion criteria, the subjects will be informed thoroughly about the experimental procedure, related considerations, possible risks, as well as benefits gain. Written informed consent will be obtained.

10.4 Experimental Procedures

(1) Explain to the study subjects, instruct the subjects to complete the following three jaw movements (including four positions) of occlusion training.

MIP: from the mandibular postural position to the most extensive and closest contact of the upper and lower dentitions, which is the maximum intercuspation position and simultaneously producing maximum bite force.

Protrusive excursion: from the intercuspation position sliding forward along the oblique direction of the anterior tangential path, and passing through the upper and lower anterior teeth opposed until it can not move forward;

Lateral movement: from the intercuspation position moves along the lingual bevel of the left canine/right canine, and passing through the upper and lower canine teeth opposed until it can not move laterally.

(2) Select the appropriate sensor based on the width and length of the maxillary arch of the subject, open the T-scan occlusion analysis software, input the width of the incisors, adjust the width of another teeth on the graph, so that the distance from the proximal to the distal of the subject is consistent. To create a personalized dental arch model.

(3) The subjects are told to sit in the dental chair in a natural position with their relaxed natural posture, and the eyes looked straight ahead to maintain the natural head position. Place the sensor in mouth, paralleling to the occlusal plane, and the centerline is aligned. Let the subjects do the maximum occlusal interdigitation and select the appropriate sensitivity according to their occlusion. The appropriate sensitivity level is the red zone with the least distribution in the yellow and blue regions, usually no more than 1-2 columns of red and orange bars.

(4) Record the image of the occlusal staggered position of the study object: guide the research subjects naturally close from the mandibular postural position to the maximum intercuspation position. At the same time, the upper and lower dentition will be bit tight until the force fades, and the corresponding image is recorded and recorded three times.

(5) Record the image of the occlusal movement of the research subject: guide the research subjects naturally close from the mandibular postural position to the maximum intercuspation position. At this time, let the research subject move the mandible forward to the anterior teeth until the edge to edge and can not move forward, record the corresponding image, repeat the record three times.

(6) Record the image of the lateral occlusal motion, guide the research subjects naturally close from the mandibular postural position to the maximum intercuspation position. At this time, let the subjects make lateral occlusions to the left and right, record the corresponding images, and repeat the recording three times.

10.5 Data collection

All measurements will be performed by one examiner.

(1) Characteristics of subject's demography, location and classification of missing single posterior teeth.

(2) Characteristics of prosthesis material and retention method, implant length, diameter, crown and implant ratio.

(3) The baseline of clinical data.

(4) The occlusal parameters of the follow up time.

10.6 Statistical Analysis

The statistical analysis method use one-way ANOVA, paired T test or Friedman test and Wilcoxon test.

11. Participant accessibility detail/ methods / medical record

11.1 Participant will be recruited from patients who have been undergone dental implant treatment at Faculty of Dentistry, Thammasat hospital according to inclusion criteria. 3 months before the study begin, the patients who have received successful single implant in the posterior teeth will be selected according to the inclusion criteria. The purpose, significance and requirements of the study will be explained to the subjects on the day of the appointment when the crown insertion. In the case of obtaining informed consent of the research subject, the research will be carried out.

11.2 The details of these patients will be obtained from the standard case record form （Appendix A)of the Implantology clinic, Faculty of Dentistry, Thammasat University. In order to judge whether it meets the inclusion criteria or not.

11.3 Details of inclusion criteria about clinical findings from extra-oral , intra oral examination and soft tissue.

11.3.1 Extra-oral examination

(1) There is no obvious trembling of the maxillofacial muscles.

(2) There are no obviously pain, friction sound and dysfunction of the temporomandibular joint.

11.3.2 Intra-oral examination

(1) Single posterior implant.

(2) No pain on function after 8-12 weeks implant placement.

(3) Implant without mobility.

(4) The radiographic crestal bone Loss is between 2 and 4 mm from initial surgery.

(5) No exudates history

(6) The color, shape and texture of the gums are normal and there are no obvious bleeding when probing natural teeth.

(7) Absence of anterior open bite.

(8) No obvious signs of tooth wear for natural teeth.

11.4 Explain the benefit of the study to patients who meet the inclusion criteria and ask for informed consent.

11.5 In the following research process, the co-investigators will keep contact to patients for follow-up. (The detailed illustration of follow-up requests has been given to patient when signed the informed consent form at the beginning of research.)

12. Research Schedule throughout the project (Timetable in each month identified all research steps including ethical review committee submitting)

Time / Plan	2019			2020				2021	
	May	Jun-Aug	Aug-Dec	Jan-Mar	Mar-Jun	Jun-Sep	Sep-Dec	Jan-Mar	Mar-Jun
Research proposal									
Ethical Approval									
Study Preparation									
Clinical Trials									
Data collection									
Manuscript Writing									
Dissertation defense									

Principal investigator's signature ....

(Zhou Ting)

13. Human Subjects protection

13.1The occlusion measurement requires patient to complete a normal occlusion exercise, it is free of any negative effects and adverse reactions.

13.2 The procedure(s) acted upon participant would be exactly the same as indicated in the information. Any of personal information will be kept confidential. Any of personal information which could be able to identify you will not appear in the report.

13.3 Participants have the right to withdraw from this research protocol at any time as he/she wish with no need to give any reason. This withdrawal will not have any negative impact upon you (eg: still receive the usual services).

13.4 If participants have any question or would like to obtain more information, the researcher can be reached at all time. If the researcher has new information regarding benefit on risk/harm, participants will be informed as soon as possible.” This practice will provide an opportunity for participants to decide whether to stay/not stay with the project.

13.5 If participants are not treated as indicated in the information sheet, they can report to The Ethical Review Sub-Committee Board for Human Research Involving Sciences, Thammasat University, No. 3 (ECScTU), Room No. 110, Piyachart Building, 1st Floor, Thammasat University Rangsit Campus, Prathumthani 12121, Thailand, Tel: 0-2986-9213 ext.7358 E-mail: ecsctu3@nurse.tu.ac.th

14. Expected Benefits

14.1 Clinicians usually evaluate the outcome based the success criterion most commonly reported in clinical reports is the survival rate, meaning whether the implant is still physically in the mouth or has been removed. As an alternative of natural teeth, the effects of Implants are self-evident. However, due to the difference between implant and natural teeth, how to maximize improve function and longevity of it is the lifelong pursuit of medical workers. It will improve the treatment outcome and protocol design by measuring and analysis of implant occlusion characteristic.

14.2 Although many clinical studies have shown high success rates with dental implant treatments, several studies have reported failures and complications for diverse reasons, in which including overloading resulting from improper occlusion. Therefore, it is necessary to analyze the implant occlusion and evaluate it regularly. This will conduce the elimination of potential risky factors and achieve longer-term stability.

14.3 At present, the clinically relies mainly on the articulating paper for the occlusion adjustment. Considering the low correlation between the articulating paper and over loading, the occlusal adjustment should be more accurate and objective under the guidance of T-scan, to achieve the better treatment outcome and balanced occlusion.

15. Bibliography

[1] Koyano, K. and D. Esaki, Occlusion on oral implants: current clinical guidelines. J Oral Rehabil, 2015. 42(2): p. 153-161.

[2] Adell R, Eriksson B, Lekholm U, Brånemark PI, Jemt T. Long-term follow-up study of osseointegrated implants in the treatment of totally edentulous jaws. Int J Oral Maxillofac Implants 1990; 5(4): 347-59.

[3] Goh, E. and L.P. Lim, Implant maintenance for the prevention of biological complications: Are you ready for the next challenge? J Investig Clin Dent, 2017. 8(4).

[4] Graves, C.V., et al., The Role of Occlusion in the Dental Implant and Peri-implant Condition: A Review. Open Dent J, 2016. 10: p. 594-601.

[5] Isidor, F., Histological evaluation of peri-implant bone at implants subjected to occlusal overload or plaque accumulation. Clin Oral Implants Res, 1997. 8(1): p. 1-9.

[6] Schwarz, M.S., Mechanical complications of dental implants. Clin Oral Implants Res, 2000. 11 Suppl 1: p. 156-8.

[7] Hsu, Y.T., et al., Biomechanical implant treatment complications: a systematic review of clinical studies of implants with at least 1 year of functional loading. Int J Oral Maxillofac Implants, 2012. 27(4): p. 894-904.

[8] Schulte, W., Implants and the periodontium. Int Dent J, 1995. 45(1): p. 16-26.

[9] Kim Y, Oh TJ, Misch CE, et al.Occlusal considerations in implant therapy:Clinical guidelines with biomechanical rationale. Clin Oral Implants Res. 2005;16:26–35.

[10] Gross MD. Occlusion in implant dentistry. A review of the literature of prosthetic determinants and current concepts. Aus Dent J. 2008;53(suppl 1):S60–S68.

[11] Adell R. Tissue integrated prostheses in clinical dentistry. Int Dent J. 1985;35:259–265.

[12] Sarfaraz, H., et al., A three-dimensional finite element analysis of a passive and friction fit implant abutment interface and the influence of occlusal table dimension on the stress distribution pattern on the implant and surrounding bone. J Indian Prosthodont Soc, 2015. 15(3): p. 229-36.

[13] Wennerberg A, Carlsson GE, Jemt T. Influence of occlusal factors on treatment outcome: a study of 109 consecutive patients with mandibular implant-supported fixed prostheses opposing maxillary complete dentures. Int J Prosthodont 2001; 14(6): 550-5.

[14] Davies S & Gray R. M. What is occlusion? Br. Dent. J. 2001 Sep 8;191(5):235-8, 241-5.

[15] Fu, J.H., Y.T. Hsu and H.L. Wang, Identifying occlusal overload and how to deal with it to avoid marginal bone loss around implants. Eur J Oral Implantol, 2012. 5 Suppl: p. S91-103.

[16] Engelman MJ, Craig JA. Clinical decision making and treatment planning in osseointegration. Implant Dent. 1997;6:68.

[17] Lundgren D, Laurell L. Biomechanical aspects of fixed bridgework supported by natural teeth and endosseous implants. Periodontol 2000. 1994;4:23–40.Rilo B, Silva JL, Mora MJ, et al.

[18] Rilo, B., et al., Guidelines for occlusion strategy in implant-borne prostheses. A review. Int Dent J, 2008. 58(3): p. 139-45.

[19] O'Mahony A, Bowles Q, Woolsey G, et al. Stress distribution in the single unit osseointegrated dental implant: Finite element analyses of axial and off-axial loading. Implant Dent. 2000;9:207–218.

[20] Esposito M, Hirsch J, Lekholm U, Thomsen P. Differential diagnosis and treatment strategies for biologic complications and failing oral implants: a review of the literature. Int J Oral Maxillofac Implants. 1999;14:473–490.

[21] Duyck J, Vandamme K. The effect of loading on peri-implant bone: a critical review of the literature. J Oral Rehabil 2014 Oct;41(10):783-794.

[22] Naert I, Duyck J, Vandamme K. Occlusal overload and bone/implant loss. Clin Oral Implants Res 2012 Oct;23 (Suppl 6):95-107.

[23] Chang M, Chronopoulos V, Mattheos N. Impact of excessive occlusal load on successfully-osseointegrated dental implants: a literature review. J Investig Clin Dent 2013 Aug;4(3):142-150.

[24] Roque, M.A., G.O. Gallucci and S.J. Lee, Occlusal Pressure Redistribution with Single Implant Restorations. J Prosthodont, 2017. 26(4): p. 275-279.

[25] Madani, A.S., et al., Post-insertion Posterior Single-implant Occlusion Changes at Different Intervals: A T-Scan Computerized Occlusal Analysis. J Contemp Dent Pract, 2017. 18(10): p. 927-932.

[26] Qadeer S, Kerstein R, Kim RJ, et al: Relationship between articulation paper mark size and percentage of force measured with computerized occlusal analysis. J Adv Prosthodont 2012;4:7-12.

[27] Carey JP, Craig M, Kerstein RB, et al: Determining a relationship between applied occlusal load and articulating paper mark area. Open Dent J 2007;1:1-7

[28] Saad MN, Weiner G, Ehrenberg D, Weiner S. Effects of load and indicator type upon occlusal contact markings. J Biomed Mater Res B Appl Biomater 2008 Apr;85(1):18-22.

[29] Kerstein R. B. T-scan III applications in mixed arch and complete arch, implant –supported prosthodontics. Dent. Implantol. Update. 2008 Jul;19(7):49-53.

[30] Montgomery M. W, Shuman L & Morgan A. T-scan dental force analysis for routine dental examination. Dent. Today. 2011 Jul;30(7):112-4, 116.

[31] Trpevska, V., et al., T-scan III system diagnostic tool for digital occlusal analysis in orthodontics - a modern approach. Pril (Makedon Akad Nauk Umet Odd Med Nauki), 2014. 35(2): p. 155-60.

[32] Garg AK: Analyzing dental occlusion for implants: Tekscan's T-Scan III. Dent Implantol Update 2007;18:65-70

[33] Lee S.M and J.W. Lee, Computerized occlusal analysis: correlation with occlusal indexes to assess the outcome of orthodontic treatment or the severity of malocculusion. Korean J Orthod, 2016. 46(1): p. 27-35.

[34] Koos, B., et al., Precision of an instrumentation-based method of analyzing occlusion and its resulting distribution of forces in the dental arch. J Orofac Orthop, 2010. 71(6): p. 403-10.

[35] Pellicer-Chover, H., et al., Influence of occlusal loading on peri-implant clinical parameters. A pilot study. Med Oral Patol Oral Cir Bucal, 2014. 19(3): p. e302-7.

[36] huder, T., et al., Association between occlusal force distribution in implant overdenture prostheses and residual ridge resorption. J Oral Rehabil, 2017. 44(5): p. 398-404.

[37] CotruTa, A.M., et al., Analyzing the morphology and intensity of occlusal contacts in implant-prosthetic restorations using T-Scan system. Rom J Morphol Embryol, 2015. 56(1): p. 277-81.

[38] Dario LJ. How occlusal forces change in implant patients: a clinical research report. J Am Dent Assoc 1995 Aug;126(8):1130-1133.

16. Curriculum vitae （Form ScF 07_02）

17. Appendix

Research instruments (such as questionnaire, interviewed guideline, evaluation form)/ experimental program/ teaching plan/ teaching manual/ training program were approved by experts as follows:

1 ….. Standard case record form (Appendix A)

2….. T-scan III bite analyzer (Appendix B)

T-scan is a digital occlusal detection tool that records and measures tooth contact, force, and timing in real-time using a thin, flexible, pressure-sensitive bite transducer embedded in a dental arch-shaped recording sensor. Not only it can reflect the position of the occlusal contact point, and the area of the contact point, the strength and

distribution of the contact point objectively, but also recording the instantaneous occlusal contact in the chewing cycle to the nearest 0.01 second dynamically, It is better than other occlusion recording methods on accuracy and sensitivity, That providing a good means of research for the quantitative study of occlusal balance. T-Scan is mainly composed of sensor, connection handle, computer and analysis software. The sensor is a thickness of 60-100 μm and placed on the occlusal surface of the subject. The diaphragm has a horseshoe-shaped sensing area with small wires arranged in a longitudinal and transverse direction. When the wire is subjected to the occlusion pressure, the current change which can be transmitted to the computer through the control device and analyzed by software occurs. After that, it is possible to detect the number of occlusal contact points and the change of the occlusal force in different parts with time. It is a quantitative, objective and reliable "dynamic" occlusal evaluation method and some scholars have used it for the analysis of implants occlusion which including morphology and density, for the relationship between excessive force and bone loss, influence of occlusal loading on peri-implant clinical parameters and so on.

3….. File about T-scan bite analyzer

Remark: The protocol is approved for one year from the approval signature date.

APPENDIX A

บร-นศ-8 (Charting)

โรคประจำตัว

บัตรผู้ป่วยทันตกรรม

Dental Chart Record

คณะทันตแพทยศาสตร์มหาวิทยาลัยธรรมศาสตร์

Faculty of Dentistry Thammasat University

ชื่อ-นามสกุล..HN..................

วันเดือนปีเกิด...อายุ........................ปี

อาชีพ...

สถานที่ทำงาน..เบอร์โทรศัพท์............

สถานภาพสมรส ☐ โสด ☐ แต่งงาน ☐ หย่า ☐ ม่าย

ที่อยู่ที่สามารถติดต่อได้

1..เบอร์โทรศัพท์..................

2..เบอร์โทรศัพท์..................

ข้าพเจ้าผู้มีนามข้างท้ายนี้ขอยอมรับการรักษาตามหลักวิทยาการ ณ สถานที่นี้ทุกประการ
แม้การนั้นจะได้กระทำโดยนักศึกษาและข้าพเจ้าขอรับรองว่า จะไม่เรียกร้องสิทธิใดๆทั้งสิ้นตามกฎหมาย

I agree to comply with the regulations of the Dental School Clinics. Permission is granted to the Dental School Faculty and students to perform procedures necessary for my therapy which will be used for teaching purposes. I understand that x-ray pictures and other records remain the property of the school and may be used for publication within the guidelines of Thammasat University Dental School.

ลงชื่อ..วันที่...............

(Signature) (Date)

แบบสอบถามประวัติการรักษาทางทันตกรรม

1. โปรดระบุความต้องการของการมารับการรักษาในวันนี้ (Chief compliant)
   ……………………………………………………………………………
2. อาการของปัญหาที่ท่านกำลังประสบอยู่ที่ต้องการได้รับการแก้ไข (Present illness)
   ……………………………………………………………………………
   ……………………………………………………………………………
3. ท่านเคยได้รับอุบัติเหตุหรือเกิดภยันตรายที่บริเวณใบหน้า ตัวฟัน หรือขากรรไกรหรือไม่
   ☐ ไม่เคย ☐ เคย โปรดระบุถึงปัญหาที่เกิดขึ้น.............……………………..
4. ประวัติการรักษาทางทันตกรรมที่ท่านเคยได้รับมาก่อน
   ☐ ไม่เคยได้รับการรักษาใดๆมาก่อนเลย (กรุณาผ่านไปตอบคำถามข้อ 7)
   ☐ เคยได้รับการรักษา ได้แก่
   - ☐ เคลือบฟลูออไรด์ ☐ เคลือบหลุมร่องฟัน
   - ☐ อุดฟัน
   - ☐ รักษาคลองรากฟัน เมื่อ......................
   - ☐ ถอนฟัน เมื่อ.....................
   - ☐ ผ่าฟันคุด
   - ☐ ขูดหินปูน/เกลารากฟัน ความถี่................. ล่าสุดเมื่อ.......................
   - ☐ ศัลยกรรมเหงือก เมื่อ........................
   - ☐ ใส่ฟัน
   - ☐ เอกซเรย์ ครั้งล่าสุดเมื่อ.........................……………………
   - ☐ อื่นๆโปรดระบุ...................................……………………...
5. สาเหตุที่ทำให้ท่านสูญเสียฟัน (กรณีที่มีประวัติการสูญเสียฟันมาก่อน)
   ☐ ฟันผุ ☐ โรคเหงือก ☐ อุบัติเหตุ ☐ อื่น ๆ (โปรดระบุ).....................
6. ท่านเคยประสบปัญหาในการรักษาทางทันตกรรมในครั้งที่ผ่านมาหรือไม่
   ☐ ไม่เคย ☐ เคย โปรดระบุถึงปัญหาที่เกิดขึ้น........……………….……
7. การดูแลสุขภาพช่องปาก
   - ชนิดของแปรงสีฟัน ☐ แปรงสีฟันไฟฟ้า ☐ แปรงธรรมดา
   - ขนแปรง ☐ อ่อนนุ่ม ☐ ปานกลาง ☐ แข็ง
   - หน้าตัดขนแปรง ☐ เรียบ ☐ อื่น ๆ (โปรดระบุ)...................................
   - ยาสีฟัน ☐ ผงยี่ห้อ................................ ☐ ครีม ยี่ห้อ................................
   - วิธีแปรงฟัน ....
   - แปรงฟันวันละกี่ครั้ง ....
   - น้ำยาบ้วนปาก ☐ ไม่ใช้ ☐ ใช้ (โปรดระบุยี่ห้อ)...................................
   - อุปกรณ์ทำความสะอาดซอกฟัน ☐ ไม่ใช้ ☐ ใช้ (โปรดระบุ)...................................
8. เคย โปรดความประสงค์ของการมารับการรักษาของท่านในวันนี้
   ☐ ต้องการรับการรักษาเฉพาะจุดที่เป็นปัญหาเท่านั้น
   ☐ ต้องการรับการรักษาแบบต่อเนื่องเพื่อแก้ไขทุกปัญหาที่มีอยู่ในช่องปาก

ลงชื่อผู้ให้ประวัติ..วันที่............….

ผู้ให้การรักษา.../อาจารย์.................................

ชื่อ-นามสกุล..HN......... บร-นศ-๘.2 (Charting)

แบบสอบถามประวัติสุขภาพทั่วไปครั้งที่ 1

โปรดทำเครื่องหมาย ✕ ในช่อง ☐ ที่ท่านต้องการตอบ

1. ในขณะนี้ สุขภาพโดยรวมของท่าน ☐ แข็งแรงดี ☐ กำลังมีปัญหาสุขภาพ
2. ในขณะนี้ท่านกำลังอยู่ภายใต้การดูแลของแพทย์ ☐ ไม่ใช่ ☐ ใช่...................
3. ในขณะนี้ท่านมียารับประทานเป็นประจำหรือไม่ ☐ ไม่มี ☐ มี ชื่อยา...............
4. ประจำเดือนครั้งล่าสุด (เฉพาะสุภาพสตรี)..
5. ท่านเคยหรือกำลังกินยาในกลุ่มต่อไปนี้หรือไม่ในช่วง 6 เดือนที่ผ่านมา

ยาในกลุ่มสเตียรอยด์	☐ ไม่มี	☐ มี	☐ ไม่แน่ใจ
ยาป้องกันการแข็งตัวของเลือด	☐ ไม่มี	☐ มี	☐ ไม่แน่ใจ
ยากล่อมประสาท	☐ ไม่มี	☐ มี	☐ ไม่แน่ใจ
ยารักษาโรคทางกระดูก	☐ ไม่มี	☐ มี	☐ ไม่แน่ใจ

6. ประวัติการแพ้ยาของท่านดังต่อไปนี้

ยาในกลุ่มเพนนิซิลิน	☐ ยังไม่เคยแพ้	☐ แพ้ยา
ยาแก้อักเสบตัวอื่น	☐ ยังไม่เคยแพ้	☐ แพ้ยา..............................
ยาแก้ปวด	☐ ยังไม่เคยแพ้	☐ แพ้ยา...............................
ยาชา	☐ ยังไม่เคยแพ้	☐ แพ้ยา

7. ท่านเคยได้รับการผ่าตัดในโรงพยาบาลมาก่อนหรือไม่ ☐ ไม่เคย ☐ เคยด้วยสาเหตุ.............
8. ท่านเคยนอนรับการรักษาในโรงพยาบาลมาก่อนหรือไม่ ☐ ไม่เคย ☐ เคยด้วยสาเหตุ.............
9. ท่านเคยหรือกำลังมีปัญหาทางด้านสุขภาพดังต่อไปนี้หรือไม่

โรคไข้รูมาตอยด์	☐ ไม่มี	☐ มี	
โรคกระดูกพรุน	☐ ไม่มี	☐ มี	
โรคหัวใจ	☐ ไม่มี	☐ มี	ระบุชื่อโรค....................
โรคความดันโลหิต	☐ ไม่มี	☐ มี	ระบุชื่อโรค....................
โรคเลือด	☐ ไม่มี	☐ มี	ระบุชื่อโรค....................
โรคปอด	☐ ไม่มี	☐ มี	ระบุชื่อโรค....................
โรคหอบหืด	☐ ไม่มี	☐ มี	ระบุชื่อโรค....................
โรคไต	☐ ไม่มี	☐ มี	ระบุชื่อโรค....................
โรคกระเพาะและลำไส้	☐ ไม่มี	☐ มี	ระบุชื่อโรค....................
โรคตับ	☐ ไม่มี	☐ มี	ระบุชื่อโรค....................
โรคเบาหวาน	☐ ไม่มี	☐ มี	ระบุชื่อโรค....................
ชัก	☐ ไม่มี	☐ มี	ระบุชื่อโรค....................
ภูมิแพ้	☐ ไม่มี	☐ มี	แพ้..
โรคภูมิคุ้มกันบกพร่อง	☐ ไม่มี	☐ มี	ระบุชื่อโรค....................
กำลังตั้งครรภ์	☐ ไม่มี	☐ มี	อายุครรภ์........เดือน
มีความพิการทางด้านอวัยวะ	☐ ไม่มี	☐ มี	ระบุ..............................
มีความพิการทางด้านสติปัญญา	☐ ไม่มี	☐ มี	ระบุ..............................

ลงชื่อผู้ให้ประวัติ...วันที่.............

ความสัมพันธ์กับผู้ป่วย...

ผู้ให้การรักษา../อาจารย์..........................

Clinical findings from extra-oral examination

General appraisal ☐ normal ☐ abnormal……………..

Skull (features, contour and size) ☐ normal ☐ deformity…………….

Facial form & profile ☐ normal ☐ others…………….

Skin
- texture ☐ normal ☐ dry ☐ others
- color ☐ normal ☐ pale ☐ redden
  ☐ hyperpigmentation ☐ others……….
- lesion ☐ type……….. ☐ localized
  ☐ generalized

Eyes ☐ normal ☐ inflammation
☐ abnormal ………

Neurologic deficit ☐ yes……….. ☐ no

Lymph nodes of head and neck ☐ normal
☐ abnormal
- ☐ enlargement…………..
- ☐ movable
- ☐ fixed…………………..

TMJ ☐ normal ☐ joint sound…….
☐ swelling……. ☐ tenderness……..

Masticatory muscles ☐ normal ☐ fatigue…………
☐ tenderness……. ☐ trismus………..

Opening pattern ☐ straight ☐ deviation……….

Range of motion ☐ normal ☐ abnormal

Salivary glands
- parotid glands ☐ normal ☐ abnormal……..
- submandibular salivary glands ☐ normal ☐ abnormal……..
- sublingual ☐ normal ☐ abnormal……..

Muscles of head and neck ☐ normal ☐ tenderness
☐ others………………

ผู้ให้การรักษา../อาจารย์.............................วันที่.....................

Clinical findings from intra-oral examination: soft tissues

Lip

- **Vermillian border** ☐ normal ☐ lesion………..
  ☐ others………………………………..
- **Lip commissure** ☐ normal ☐ lesion ………..
  ☐ others………………………………..
- **Inner surface of the lip** ☐ normal ☐ lesion ………..
  ☐ others………………………………..
- **Lip line** ☐ High lip line ☐ Gummy smile
  ☐ Low lip line

Buccal mucosa ☐ normal ☐ linear alba ☐ others……

Salivary flow rate ☐ normal ☐ dried mouth ☐ others……

Oropharynx

- **Anterior tonsillar pillar** ☐ normal ☐ others………..
- **Posterior tonsillar pillar** ☐ normal ☐ others………..
- **Palatine tonsil** ☐ normal ☐ others………..
  ☐ inflammation ☐ others………..
- **Uvula** ☐ normal ☐ others………..

Soft palate ☐ normal ☐ others………..

Hard palate ☐ normal ☐ others………..

Tongue ☐ normal ☐ geographic
☐ fissured ☐ others………..

Floor of mouth ☐ normal ☐ others………..

Periodontal status

Gingiva

……………………………………………………………………………………………
……………………………………………………………………………………………………
……………………………………………………………………………………………………
……………………………………………………………………………………………………

Gingival biotype ☐ Thick ☐ thin
Gingival recession ☐ no ☐ yes………….……………
Gingival IDP ☐ present ☐ reduce ☐ loss

Frenum attachment ☐ normal ☐ high

Local factor of periodontal disease

1. **Local factors**
   a. Plaque(O' Leary) ☐ good (<20%) ☐ fair (20-50%) ☐ poor (>50%)
   b. Calculus (supra/subgingival)
   c. Anatomical factors………………………………………………………..…………
   d. Caries related……………………………………………………….....……….
   e. Improper restoration ………………………………..…………………………
   f. Improper embrasure…………………………………………………………….
   g. Food impaction ……………………………………………………………….
   h. Prosthesis/Appliance ………………………………………………………….
   i. Others ………………………………………………………………………….
2. **Risk factors**
   a. Systemic disease………………………………………………………….
   b. Familial heredity/Genetics…………………………………………………….
   c. Drugs induces…………………………………………………………………
   d. Smoking ………………………………cigarettes/day………………pack-year
   e. Stress level (scale 1-10) ………………………………………………………
   f. Others …………………………………………………………………………
3. **Occlusal trauma** ☐ no ☐ yes……………….……..
4. **Nutritions** ☐ normal ☐ malnutritions…………………………..
   ☐ favorite food intake …………………………………………
5. **Parafunction habits** ☐ no ☐ yes……………………..
6. **Diagnosis of periodontal disease** …...
7. **Prognosis**
   overall prognosis ☐ Good ☐ Fair ☐ Poor
   individual prognosis

[illegible]

	18	17	16	15	14	13	12	11	21	22	23	24	25	26	27	28
**Good**																
**Fair**																
**Poor**																
**Questionable**																
**Hopeless**																
**Hopeless**																
**Questionable**																
**Poor**																
**Fair**																
**Good**																
	48	47	46	45	44	43	42	41	31	32	33	34	35	36	37	38

8.Treatment plan

Systemic phase

……………………………………………………………………………………………
……………………………………………………………………………………………
……………………………………………………………………………………………

Hygienic phase

……………………………………………………………………………………………
……………………………………………………………………………………………
……………………………………………………………………………………………

Corrective phase

……………………………………………………………………………………………
……………………………………………………………………………………………
……………………………………………………………………………………………

Maintenance phase

……………………………………………………………………………………………
……………………………………………………………………………………………
……………………………………………………………………………………………

ผู้ให้การรักษา../อาจารย์.............................วันที่.....................

Dental implant examination

Tooth no./Area of implantation ……………………………………………………………………
Alveolar ridge width B-L………………mm
M-D……………...mm (at gingival margin)
Approximate tissue thickness……………mm
Interocclusal space………………………mm
Lateral movement ☐ canine guidance ☐ group function
Type of antagonist tooth ☐ natural ☐ prosthesis……………

Radiographic finding
Bone available from sinus floor to Alv. Crest………………………..mm.

from Inf Alv.canal to Alv. Crest………………………..mm.
M-D width of neighboring tooth at CEJ ……………………………………..mm.
CEJ to Alv. Crest………………………..mm.

Operation☐ Consent form signed
a. Preparation & Operative finding :
Anesthesia: ☐ local ☐ general
Bone support: ☐ sufficiency
☐ Horizontal bone defect ☐ Vertical bone defect
Bone shape: ☐ knife edge ☐ round
☐ dehiscence ☐ fenestration

Bone augmentations
☐ Primary augmentation before implant placement
☐ vertical bone graft ☐ horizontal bone graft
☐ onlay bone graft ☐ sandwich technique
☐ distraction ☐ others………………………………..
☐ GBR simultaneously with implant placement

☐Sinus lift: ☐ closed (Osteotome) ☐ opened (lateral window)

Augmentation material: ☐ Autogenous ☐ Xenograft ☐ Synthetics ☐ others……………..
Membrane: ☐Bio-Gide ☐ Biomend ☐ others………………………………………...
Pin: System……………….size………………

b. Treatment :
Implant system:
☐Straumann ☐Astra Tech ☐Noble Biocare ☐Zimmer ☐Osstem
☐others………
Configuration:
☐Tissue level ☐Bone level ☐Taper ☐Straight
☐others…………………...……………………
Irrigation: ☐ external ☐ internal
Bone quality Type 1 – 2 – 3 - 4
Drilling speed ……………………………...…………………………………. rpm
Implant insertion speed ……………………………………..…………Ncm

Implant location…#…………
Implant size: ☐Ø………………..……..mm. ☐length ……………….. mm.
Cover screw/Healing abutment : ☐ Height ………… mm.☐Ø……………..mm.

Implant location…#…………
Implant size: ☐Ø…………….……..mm. ☐ length ……………………… mm.
Cover screw/Healing abutment : ☐ Height …………… mm.☐Ø……………..……mm.

Implant location…#…………
Implant size: ☐Ø…………….……..mm. ☐ length ……………………… mm.
Cover screw/Healing abutment : ☐ Height …………… mm.☐Ø……………..……mm.

Implant location…#…………
Implant size: ☐Ø…………….……..mm. ☐ length ……………………… mm.
Cover screw/Healing abutment : ☐ Height …………… mm.☐Ø……………..……mm.

Implant location…#…………
Implant size: ☐Ø…………….……..mm. ☐ length ……………………… mm.
Cover screw/Healing abutment : ☐ Height …………… mm.☐Ø……………..……mm.

Implant location…#…………
Implant size: ☐Ø…………….……..mm. ☐ length ……………………… mm.
Cover screw/Healing abutment : ☐ Height …………… mm.☐Ø……………..……mm.

Implant location…#…………
Implant size: ☐Ø…………….……..mm. ☐ length ……………………… mm.
Cover screw/Healing abutment : ☐ Height …………… mm.☐Ø……………..……mm.

Implant location…#…………
Implant size: ☐Ø…………….……..mm. ☐ length ……………………… mm.
Cover screw/Healing abutment : ☐ Height …………… mm.☐Ø……………..……mm.

Implant location…#…………
Implant size: ☐Ø……….……….mm. ☐ length …………………….. mm.
Cover screw/Healing abutment : ☐ Height ………..mm. ☐Ø……….……mm.

Esthetic type: ☐ yes ☐ no
Stage: ☐ one (transmucosal) ☐ two (submerged)
Loading: ☐ immediate ☐ early ☐ delayed
Medication: ☐ pre-medication ……………………………………………………
☐ post-medication ……………………………………………..……….
☐ chlorhexidine mouthwash ………..…………………………………….

c. Complication
☐ intra – operation …………………………………………………………………
☐ immediate post – operation ……………………………..……………………….
☐ infection ☐ swelling ☐ pus exudates
☐ loose of implant ☐ wrong direction
☐others……………………………………………………………………………

บร-นศ-8.6(Charting)

แบบตรวจช่องปาก คณะทันตแพทยศาสตร์ มหาวิทยาลัยธรรมศาสตร์

	18	17	16	15	14	13	12	11	21	22	23	24	25	26	27	28	
Vitality																	Vitality
Palpation/ Percussion																	Palpations/ Percussion
Mobility																	Mobility
BOP/Suppuration																	BOP/Suppuration
CAL																	CAL
PD																	PD
Gingival margin																	Gingival margin
Attached gingiva																	Attached gingiva

Buccal

Palatal

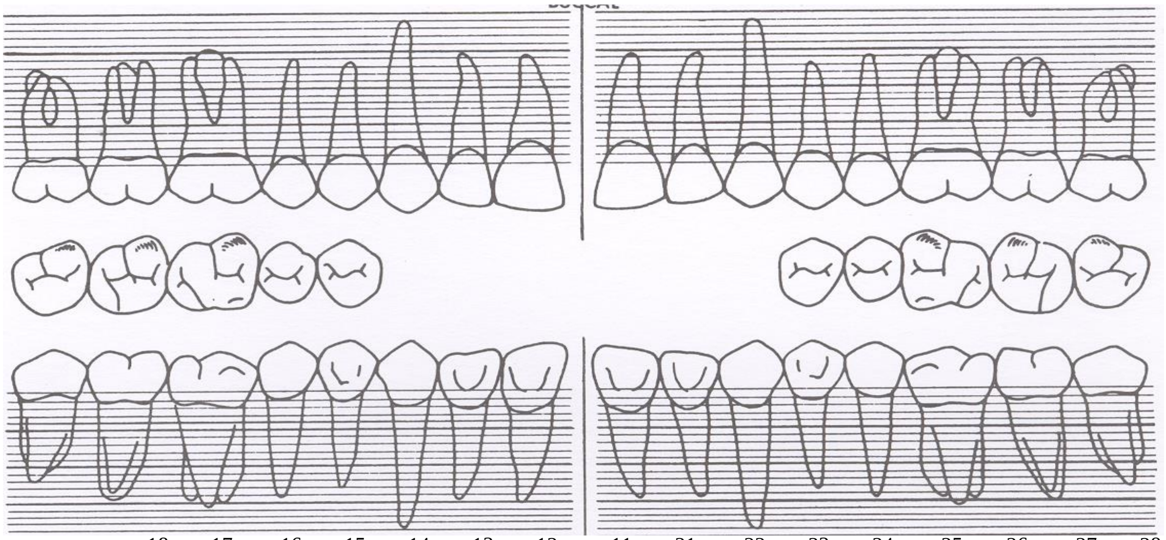

Buccal

Palatal

	18	17	16	15	14	13	12	11	21	22	23	24	25	26	27	28	
Attached gingiva																	Attached gingiva
Gingival margin																	Gingival margin
PD																	PD
CAL																	CAL

CAL																	CAL
PD																	PD
Gingival margin																	Gingival margin
Attached gingiva																	Attached gingiva

48 47 46 45 44 43 42 41 31 32 33 34 35 36 37 38

Lingual

Buccal

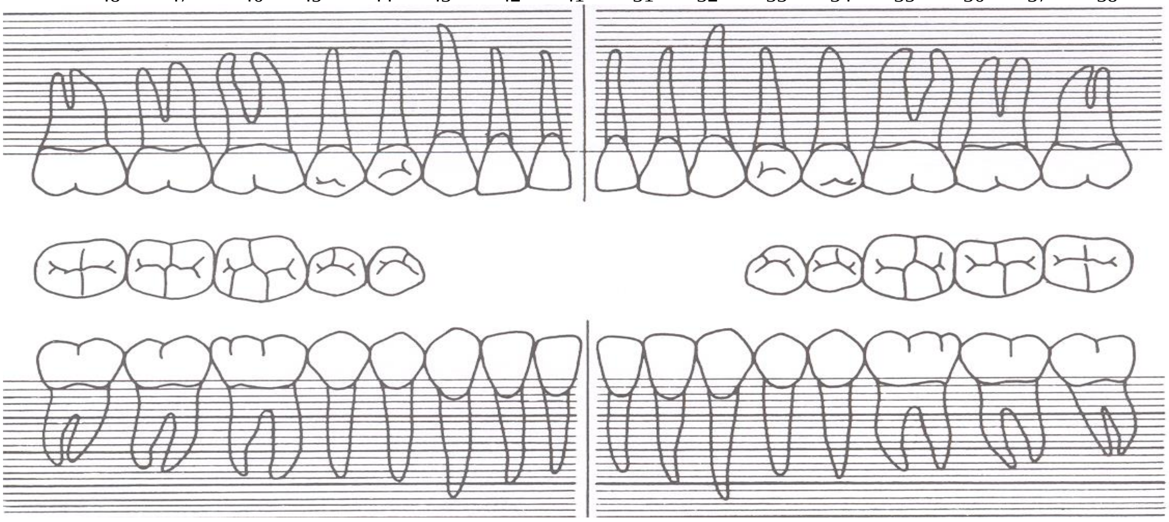

Lingual

Buccal

Attached gingiva																	Attached gingiva
Gingival margin																	Gingival margin
PD																	PD
CAL																	CAL
BOP/Suppuration																	BOP/Suppuration
Mobility																	Mobility
Palpations/ Percussion																	Palpations/ percussion
Vitality																	Vitality
	48	47	46	45	44	43	42	41	31	32	33	34	35	36	37	38	

ชื่อ-นามสกุล..HN…………………

Molar relationship:

Right ☐ class I ☐ class II ☐ class III ☐ unable to classified

Left ☐ class I ☐ class II ☐ class III ☐ unable to classified

Posterior crossbite ☐ right ☐ left ☐ no

Traumatic occlusion ☐ no ☐ yes on tooth #....................................

Canine relationship:

Right ☐ class I ☐ class II ☐ class III ☐ unable to classified

Left ☐ class I ☐ class II ☐ class III ☐ unable to classified

Anterior relationship:

Overjet……….mm. Overbite…………….mm.

Anterior crossbite ☐ no ☐ yes - tooth #……………….

Traumatic occlusion ☐ no ☐ yes - tooth #.........................

Midline:

Upper ☐ normal ☐ deviate……..mm to the……….

Lower ☐ normal ☐ deviate……..mm to the……….

Space condition:

☐ adequate ☐ excess ☐ inadequate ☐ need space analysis

ผู้ให้การรักษา../อาจารย์.............................วันที่.....................

ประวัติการส่งถ่ายภาพเอกซเรย์

วันที่-เดือน-พ.ศ. ที่ส่งถ่าย	บริเวณที่ส่งถ่าย	ชนิดของภาพถ่ายเอกซเรย์	ผู้ตรวจ/อาจารย์

Treatment rational for ……………………….HN………………. **ตรวจครั้งที่……………**

**Tooth number**	**Clinical examination**	**Radiographic interpretation**	**Diagnosis**	**Tx. options**

ผู้ให้การรักษา……………………………………**วันที่**…………………

อาจารย์……………………………………**วันที่**…………………

Treatment plan for……………………………HN……………. ตรวจครั้งที่...............

Visit	Tooth number	Procedure	Fee	Date completed	Instructor signature

ผู้ให้การรักษา...วันที่.....................

อาจารย์..วันที่.....................

APPENDIX B

T-Scan system

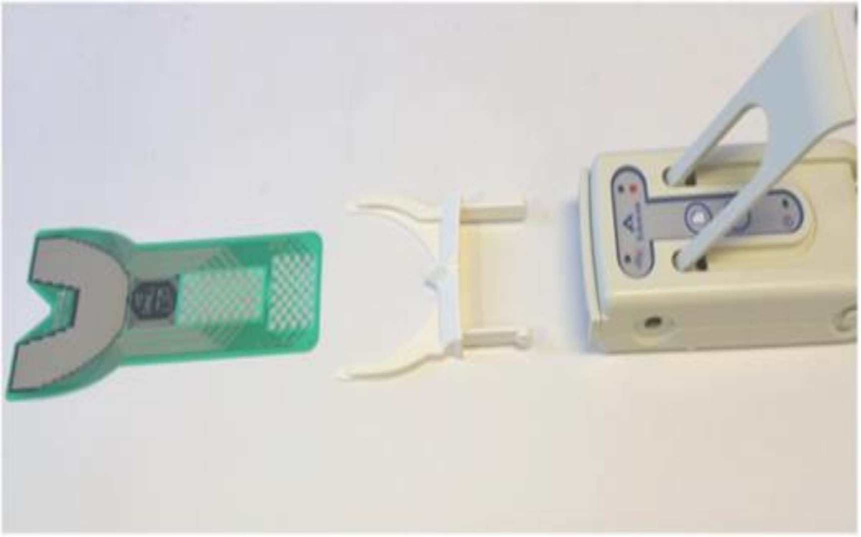

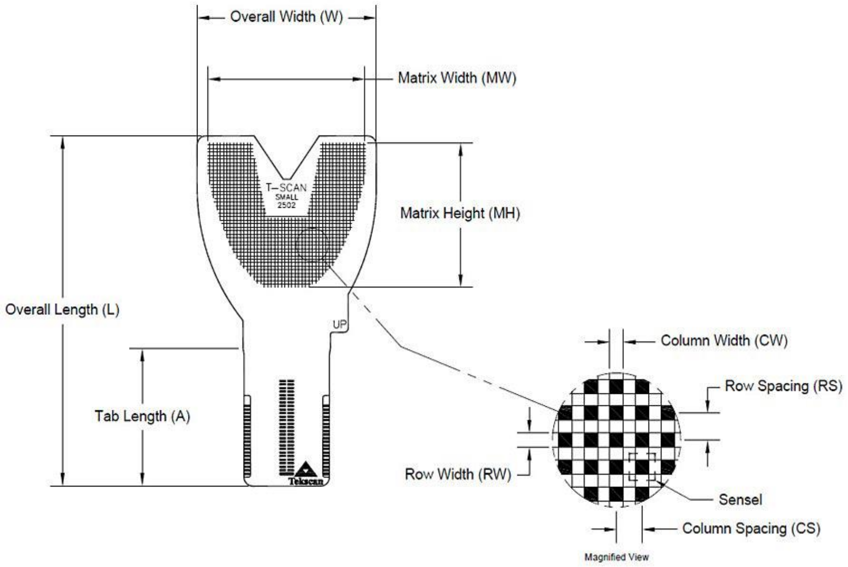

Sensor 2502 Shown
